# The impact of packaging and messaging on adherence to malaria treatment: Evidence from a randomized controlled trial in Uganda

**DOI:** 10.1016/j.jdeveco.2018.04.008

**Published:** 2018-09

**Authors:** Jessica Cohen, Indrani Saran

**Affiliations:** a Harvard T.H. Chan School of Public Health and J-PAL, Building 1, Room 1209, 665 Huntington Avenue, Boston, MA 02115, USA; b Harvard T.H. Chan School of Public Health, Building 1, 665 Huntington Avenue, Boston, MA 02115, USA

**Keywords:** Adherence, Malaria, Infectious diseases, Health behavior, Patient beliefs

## Abstract

Despite substantial public and private costs of non-adherence to infectious disease treatments, patients often do not finish their medication. We explore adherence to medication for malaria, a major cause of morbidity and health system costs in Africa. We conducted a randomized trial in Uganda testing specialized packaging and messaging, designed to increase antimalarial adherence. We find that stickers with short, targeted messages on the packaging increase adherence by 9% and reduce untaken pills by 29%. However, the currently used method of boosting adherence through costly, specialized packaging with pictorial instructions had no significant impacts relative to the standard control package. We develop a theoretical framework of the adherence decision, highlighting the role of symptoms, beliefs about being cured, and beliefs about drug effectiveness to help interpret our results. Patients whose symptoms resolve sooner are substantially less likely to adhere, and the sticker interventions have the strongest impact among these patients.

## Highlights

• Non-adherence to malaria medication has substantial public and private costs.
• We tested the impact of drug packaging design on adherence to malaria medication.
• We conducted a randomized field experiment with 2641 households in Uganda.
• Stickers on packages with short, targeted messages increased adherence by 9%.
• Sticker messages have larger impact among patients with early symptom resolution.

## 1 Introduction

With over two hundred million cases a year and nearly half a million deaths, malaria remains a primary cause of morbidity, health system costs, and lost productivity in Africa (WHO, 2015; WHO, 2010a; Barofsky et al., 2015; Fink and Masiye, 2015; Dillon et al., 2014). Over the past 15 years, however, malaria deaths have declined by nearly 50 percent (WHO, 2015), largely because of increased coverage of prevention and treatment technologies (Bhatt et al., 2015). For example, insecticide-treated nets (ITNs) to prevent malaria, and artemisinin-combination therapies (ACTs) to treat the disease, are highly effective technologies when they are widely accessible and properly used. However, the health benefits of ITNs are diluted when people do not sleep under them consistently, and the impact of ACTs is limited by patient non-adherence to the treatment regimen. Despite the fact that ACTs are only a three day treatment, in some contexts up to 60% of patients do not finish the full course of drugs (Banek et al., 2014). Incomplete treatment with ACTs increases the probability of remaining parasites (Stepniewska et al., 2010; Muhindo et al., 2014; Das et al., 2013) and of malaria recurrence (Beshir et al., 2013; Stepniewska et al., 2010), which can be costly to the patient in terms of additional treatment and lost productivity. There are also public costs of non-adherence including an increased burden on health systems, potential externalities from disease transmission (Gersovitz and Hammer, 2004), and greater risk of the emergence and spread of resistant forms of the malaria parasite (White et al., 2009).^2^ Resistance to artemisinin, the primary component of ACTs, has already been identified in parts of Southeast Asia and widespread resistance to the drug would pose a major threat to malaria control efforts (Ashley et al., 2014; White, 2012; Slater et al., 2016).

This paper explores adherence to ACTs among patients seeking malaria treatment in the private sector. The private health sector in sub-Saharan Africa encompasses a wide variety of outlet and provider types, ranging from small informal shops to more formal private health centers, and is the source of malaria treatment for more than 40 percent of patients (WHO, 2014). While private outlets are typically more accessible than public sector facilities (closer proximity, open longer hours, etc), the quality of medical advice and product varies widely and is often substandard. In addition to ACTs, most private outlets offer a variety of older, less effective antimalarials, and few offer a malaria diagnostic test (Littrell et al., 2011; Poyer et al., 2015). Counterfeit anti-malarial drugs are also prevalent in this region (Bjorkman-Nyqvist et al., 2013; Nayyar et al., 2012). As others have noted, decisions about malaria treatment are thus occurring in a very noisy learning environment (Bjorkman-Nyqvist et al., 2013; Cohen et al., 2015a; Adhvaryu, 2014), and patients may face substantial uncertainty about the relative effectiveness of different anti-malarial drugs, how to take them properly, and about whether the illness being treated is even truly malaria. Previous studies have tested ways to improve malaria treatment in this context by increasing the availability of malaria rapid diagnostic tests, which can improve the targeting of ACTs (Ansah et al., 2015; Awor et al., 2014; Cohen et al., 2015a, 2015b; Mbonye et al., 2015). This study explores approaches to increasing adherence to ACTs through specially designed packaging and messaging.

We conducted a randomized controlled trial in Luwero, Uganda, an area of very high malaria prevalence, in which 2641 households were given access to subsidized ACTs at local drug shops. We found that 35 percent of patients did not complete the full ACT treatment course, with similar rates of non-adherence for young children, who are at highest risk of severe morbidity and mortality from malaria infection. We experimented with several ACT packages designed to increase adherence to the medication. We first tested a current approach to boosting ACT adherence rates, used by Ministries of Health and social marketing organizations in several African countries. This specialized packaging, which we refer to as the “CAPSS” package, has pictorial instructions for illiterate patients, and a colorful, glossy design. The package aims to increase adherence by improving comprehension of dosing (using pictures) and by signaling that the drugs are high quality. We found that, despite raising the production cost of the drug by 10–50 percent, this approach had no significant effect on adherence. We also tested the impact of two inexpensive stickers affixed to the standard ACT package with short, targeted messages about adherence. Both stickers highlighted the importance of adherence and encouraged patients to finish all pills. One of the messages focused on how non-adherence reduces the likelihood that the illness is cured, while the other message emphasized that saving pills can be harmful for the community. We found that both stickers increased treatment completion by roughly 6 percentage points (9 percent) and reduced the number of remaining pills by 29 percent. Although the CAPSS package also includes messaging about the importance of adherence, this information is buried in a substantial amount of other content. This suggests that the way the information is presented on the stickers may be important for adherence – perhaps by making adherence more salient – although we do not have direct evidence of this particular mechanism.

We apply a theoretical framework of the patient adherence decision, based on patients' mid-treatment symptom severity, to help interpret patterns of adherence behavior. We find that adherence is correlated with mid-course symptom severity, beliefs about the effectiveness of ACTs, and beliefs about being cured prior to treatment completion. While we do not know whether there is a relationship between mid-course symptom severity and true cure rates, we show that there is no correlation between mid-course symptom severity and malaria parasitemia at the start of treatment, and the medical literature indicates that symptom severity is not a good indicator of parasite clearance (Makanga et al., 2011). We show that the stickers improved adherence largely among patients who reported that their symptoms resolved mid-treatment and patients who believed that their illness was cured early in the treatment course. Overall, our evidence is consistent with the possibility that the stickers caused patients to rely less on symptoms in their decision about when to stop taking the medicine. Although our study was not powered to detect potential impacts on malaria transmission, we use published estimates of the impact of adherence on malaria cure rates to show that these simple stickers cost approximately $1-$4 per averted malaria infection.

Our paper contributes to the previous literature in several ways. First, while there is a large literature in medicine and public health on the prevalence of non-adherence across disease types (Osterberg and Blaschke, 2005; Brown and Bussell, 2011; Sabate, 2003) and a growing number of interventions designed to increase medication adherence (Haynes et al., 2008; McDonald et al., 2002; Nieuwlaat et al.; van Dulmen et al., 2007), virtually no literature formalizes the adherence decision. Our paper considers how patients taking short course treatments such as ACTs and antibiotics, in the absence of reliable medical information, may use their symptoms to form beliefs about the treatment and make decisions about adherence. Our model is most similar to that of Egan and Philipson (2014) where adherence to therapies for long-term chronic conditions is conceived of as an optimal stopping problem for the patient as they learn about the value of treatment. However, for malaria, there are fewer opportunities for learning about the health benefits of the drug because the treatment course is very short and diagnostic testing is uncommon.

Second, we add to the growing literature on interventions to increase adherence, which include training of pharmacists, patient counseling, verbal instructions to patients, special reminder pill packaging (McDonald et al., 2002; Haynes et al., 2008; Bruxvoort et al., 2014), text message reminders (Raifman et al., 2014; Pop-Eleches et al., 2011), and financial incentives (Kimmel et al., 2012; Giuffrida and Torgerson, 1997; Volpp et al., 2008; DeFulio and Silverman, 2012). Most of these are tacitly built on the assumption that people *want* to adhere but face obstacles in doing so–for example, they forget to take pills, do not understand how to take pills, or face time inconsistency problems. We test interventions that target some of the reasons patients may choose non-adherence, for example because they believe they are cured or because they want to save pills for future illness episodes.

Our paper also contributes to the economics literature on treatment-seeking behavior in developing countries, in particular the central role played by the private sector, where the quality of medical advice and treatment varies greatly, and where diagnostic testing and continuity of care are very limited (Banerjee et al., 2004; Cohen et al., 2015a; Das et al., 2008; Leonard, 2013; Leonard and Masatu, 2007). Our results should have relevance beyond malaria; in particular, there are similarities to treatment-seeking for bacterial infections (such as pneumonia) where non-adherence to short course antibiotics is also a serious public health concern (Kardas, 2002; Llor et al., 2013).

Finally, we build on the literature exploring when and what types of information influence people's health behaviors. While some studies find that people respond to health-related information (Dupas, 2011; Fitzsimons et al., 2013; Jalan and Somanathan, 2008; Madajewicz et al., 2007; Thornton, 2008), others find limited impact of information on health behaviors (Kremer and Miguel, 2007; Jamison et al., 2013; Luo et al., 2012). The degree to which information affects health behaviors likely depends not only on the information content, and whether it changes people's subjective beliefs (Delavande and Kohler, 2012; Oster et al., 2013; Godlonton and Thornton, 2013; Paula et al., 2013; Thornton, 2012; Boozer and Philipson, 2000), but also on how the information is presented. For example, there is evidence that, for some preventive health behaviors, emphasizing the benefits is more effective than highlighting the costs of not doing the behavior (Gallagher and Updegraff, 2012; Rothman et al., 2006). Other research suggests that there may be a tradeoff in message effectiveness between additional information content and the length of the message (Pop-Eleches et al., 2011; Raifman et al., 2014).

The remainder of the paper proceeds as follows: Section 2 provides a theoretical framework of the adherence decision, highlighting some of the potential reasons for non-adherence. Section 3 describes the experimental design and interventions tested. In Section 4 we present the results of our intervention, and in Section 5 we use the theoretical framework to explore patterns of non-adherence in our data and how they are related to the interventions we tested. In Section 6 we estimate the cost-effectiveness of the sticker interventions. Section 7 concludes.

## 2 Theoretical framework

In this section we present a simple two-period model of the patient's adherence decision that considers several sources of uncertainty (a more detailed model is presented in A). Patients may be uncertain about whether their illness is actually malaria, since many of them are not formally diagnosed prior to starting treatment. They may also be uncertain about the effectiveness of ACTs for curing malaria, since many types of antimalarials of varying efficacy are available in public and private health markets. Lastly, patients face uncertainty about when their malaria is cured during ACT treatment. ACTs bring parasite loads down and relieve symptoms quickly (White et al., 1999), and many patients report feeling substantially better after the first few doses. However, clinical studies have found that malaria cure rates are 10–30 percentage points higher when patients take the full six doses of the drug instead of only four doses (Makanga et al., 2006; Vugt et al., 1999) and incomplete dosing is associated with recurrence of infection (Stepniewska et al., 2010; Muhindo et al., 2014; Beshir et al., 2013).^3^ While many patients will be cured before finishing the full course of ACTs, the patient cannot know with certainty when he is cured, and the difficulty in identifying ex-ante who would require less than a full dose leads to the recommendation that all patients complete the full treatment (WHO, 2010b).^4^

We consider a two-period adherence decision in which, in period one, a patient is hit with an illness shock that he believes is malaria and begins taking medication.^5^ In period two the patient decides whether to finish taking the pills or to stop treatment. The patient faces a tradeoff between the benefits of being cured of the disease and the costs of adhering to the medication. The benefit of adherence is the utility of being healthy, including productivity and wage benefits as well as the intrinsic value of good health. Patients may also value the positive externalities of being cured. The cost of adherence includes factors such as side effects, the effort required to remember to take pills, and the opportunity cost of consuming pills that could otherwise be used to treat future malaria episodes. When a patient adheres (i.e. goes on to finish the medication in period two), he guarantees that he will have the benefits of good health, but he incurs the cost of adhering. Since there is some probability that he is already cured after the first few doses he has taken in period one, it is possible that he is paying the cost of adherence unnecessarily. On the other hand, if he does not adhere, he faces some probability of continuing to suffer from malaria.

**Fig. 1:**
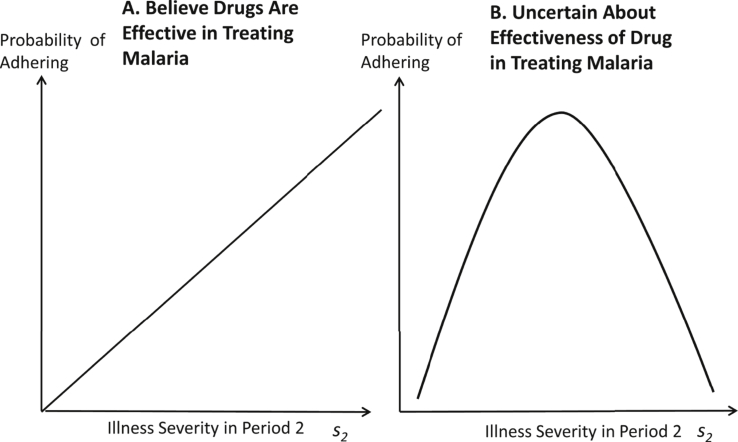
Theoretical Relationship between Mid-Course Symptom Severity and ACT Adherence by Beliefs in Drug Effectiveness. *s*_2_ is the severity of symptoms experienced in period 2 (on the second day of treatment).

We assume that the subjective probability of still having malaria is increasing with mid-course symptom severity–that is, the better the patient feels partway through treatment, the more likely he is to believe he is cured. We take symptom severity after period one as exogenous to adherence since, in our data, nearly all patients take the first few doses of treatment properly (i.e. period one adherence is nearly perfect). If the patient believes that the medication he is taking is effective (so that adhering definitely will cure him) then the patient will choose to adhere if the belief that he still has malaria in period two exceeds a threshold value that is increasing in the cost of adherence and decreasing in the utility of being healthy. Patients are thus more likely to adhere when mid-course symptom severity is high, when the costs of adhering are low (few side effects, low value of saved pills, etc.), and when the benefit to being healthy is high (see Fig. 1A).

Now consider the case where the patient is unsure about the effectiveness of these drugs (but still believes the illness is malaria). We assume that the belief that the drugs are effective is a decreasing function of mid-course symptom severity (i.e. the sicker the patient feels mid-course, the less likely he is to believe the drugs are working). This reduces the expected benefit of adherence. In this case, there is a non-linear relationship between the probability of adhering and symptom severity (see Fig. 1B). Patients who feel much better mid-course are more likely to believe the drugs are effective but also more likely to believe they are already cured and thus the expected value of adhering for them is low. Patients who still feel very sick mid-course are more likely to believe that they still have malaria, but also to believe the drugs are ineffective, so the expected utility of adhering for these patients is also low. The expected utility of adhering is therefore maximized at intermediate levels of symptom severity in the second period. In A, we consider the case where patients are uncertain about whether the illness is malaria, and show that, among these patients, we also expect adherence to be highest among those who are still moderately ill mid-way through treatment.

This theoretical framework highlights potential drivers of the adherence decision and suggests that the relationship between adherence and symptom severity could be influenced by beliefs about drug effectiveness. We explore these potential drivers of adherence in Section 5 below and also examine whether the impact of our interventions varies by these patient characteristics.

## 3 Study design and data collection

### 3.1 Experimental design and data collection

The study took place in Luwero district, located in Uganda's central region, between November 2010 and September 2011.^6^ Despite its proximity to the capital city of Kampala (about 68 km), Luwero district is rural and poor, with the majority of households engaged in subsistence farming. Luwero has a high level of malaria endemicity, with an average of over 100 infective bites per person per year (U. B. of Statistics (UBOS), 2010). The study area constitutes the catchment areas surrounding nine drug shops that were located in and around three small trading centers in the east of the district. Two of the trading centers (Busiika and Zirobwe) each had four participating drug shops, while the third trading center (Wabitungu) had the remaining one.^7^

**Fig. 2:**
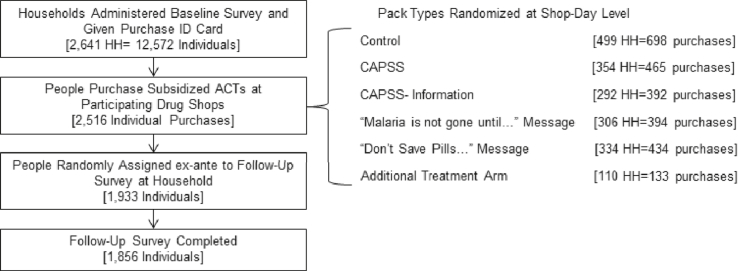
Experimental Design and Sample Sizes. The ‘Additional Treatment Arm’ is not explored in this paper since it had to be dropped early in the study to accomodate the budget. As a result, the sample size was insufficient to detect any treatment effects. People could buy ACTs multiple times over the study period.

**Fig. A1:**
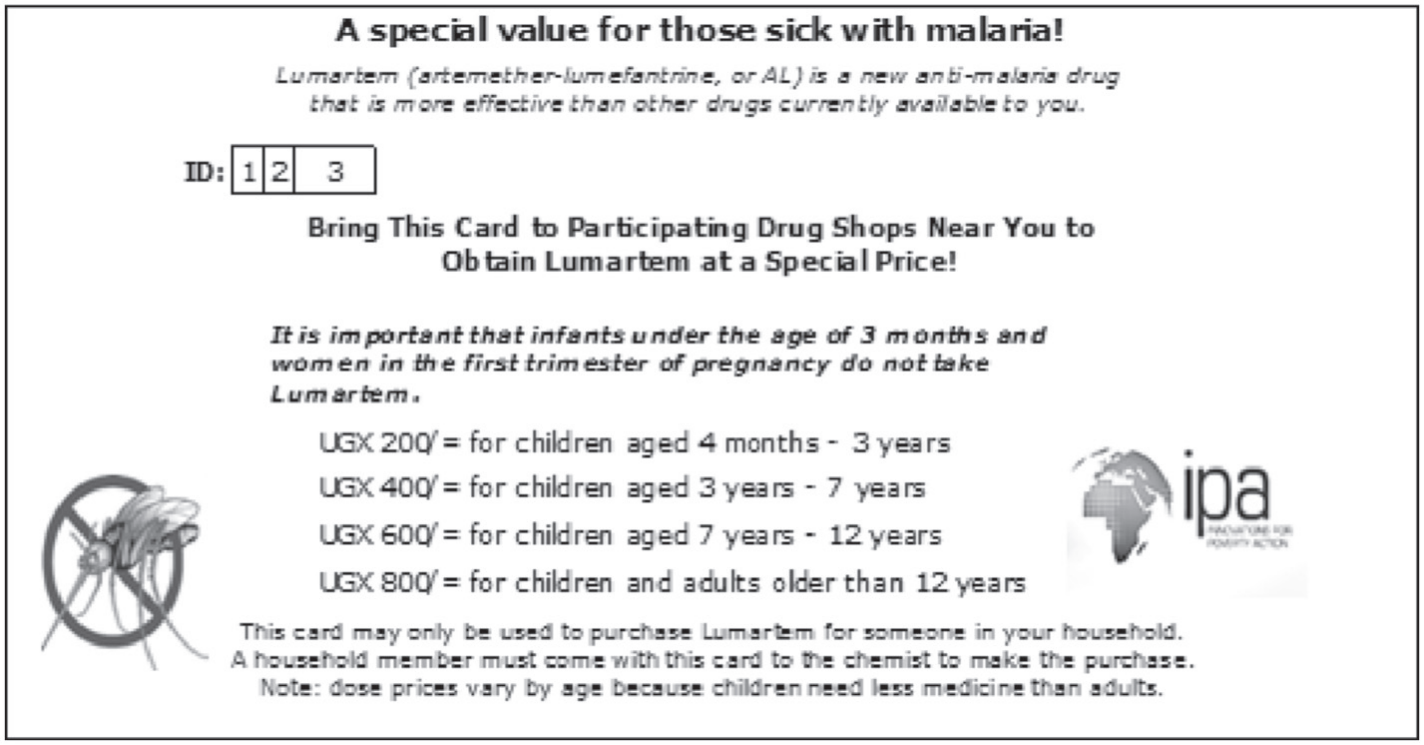
Example of Purchase ID card. Each household was given only one Purchase ID card that could be used by any member of the household as many times as they needed. The Purchase ID was used to link the drug shop and follow-up surveys for individuals to the baseline information for the household.

The experimental study design is illustrated in Fig. 2. A household census was conducted in catchment areas of roughly 2.5 km (approximately 1 h walking distance in each direction) around each shop. In November and December 2010, a team of enumerators traveled to each household in the study area to enroll participants and conduct a baseline survey. Households were then given a Purchase ID card (see Appendix Fig. A1), which enabled any household member to purchase heavily subsidized ACTs at any of the nine participating drug shops. No restrictions were placed on the number of times the card could be used during the study period and no expiration date was given.^8^ 2641 households and 12,572 individuals were enrolled in the study at baseline.^9^

The project was designed to assess the impact of various forms of ACT packaging and short messages on adherence (we define this outcome in detail below). To evaluate this, we randomized the type of packaging available at the shop-day level. An ex-ante schedule was laid out using a random number generator that indicated that Shop 1 got package A on March 1, package B on March 2, and Shop 2 got package C on March 1, etc. Surveyors assigned to each shop brought the control or treatment packs for that particular day with them, and both the study team and shop owners were blinded to the treatment assignment until the day of sale. Prior to the intervention, participating drug shop owners received a training session led by an Ugandan Ministry of Health official on storage and appropriate use of Lumartem (a brand of Artemether Lumefantrine (AL), manufactured by Cipla), the type of ACT used in this study.^10^ Attendants were instructed to follow their normal prescribing protocol for Lumartem and other anti-malarials. If the patient arrived at one of our participating shops with a Purchase ID card and wanted to buy Lumartem, they were sent to our survey team member, who sat at a table in the shop to check IDs, dispense the Lumartem in the appropriate packaging and administer a short survey, described below.

Adherence was assessed through follow-up visits to the home of the patient roughly three days after the time of ACT purchase. Not all patients received a follow-up visit: 75 percent of households were randomly assigned ex-ante to receive a follow-up visit if any member of the household purchased ACTs.^11^ Among patients who purchased ACTs, and who were members of households assigned to receive a follow-up survey, 96 percent were successfully reached for a follow-up visit. Individuals were not told of the intent to follow up in order to avoid influencing behavior, but an additional round of informed consent was sought at the time of follow-up. To further limit Hawthorne effects, enumerators asked to see the medication blisterpack and packaging in order to check the lot number, expiration date and other quality control measures, rather than to explicitly count the number of pills.

**Fig. A2:**
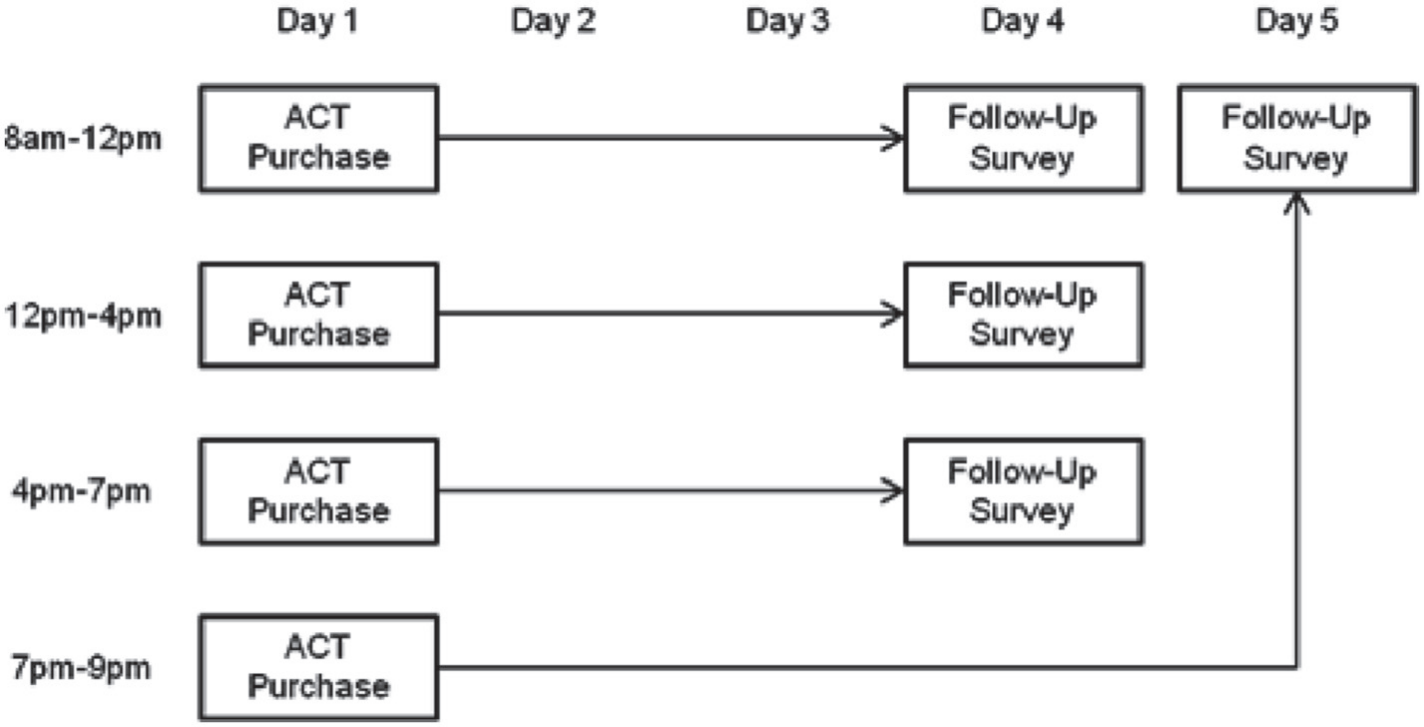
Timing of follow-up survey relative to time of ACT purchase. The follow-up survey was planned for 72 h after the time of the ACT Purchase. If people purchased ACTs after 7pm in the evening, the follow-up was scheduled for approximately 85 h later, that is the following day at 8am in the morning.

Lumartem is a six-dose treatment (with the number of pills per dose varying by age) intended to be taken over three days. The subsidized ACT price depended on the age of the patient and ranged from 200 to 800 Ugandan Shillings (approximately $0.09-$0.35 at the time of the study; see Appendix Table A1 for dosing details).^12^ The follow-up survey was scheduled for 72 h after the time of the ACT purchase unless this time fell at night, in which case the interview occurred first thing on the following morning. The timing was designed so as to allow patients sufficient time to have completed their medication while minimizing the risk that they would have already disposed of their blisterpacks.^13^ Appendix Fig. A2 describes the follow-up window in more detail.

### 3.2 Treatment arms

**Fig. 3:**
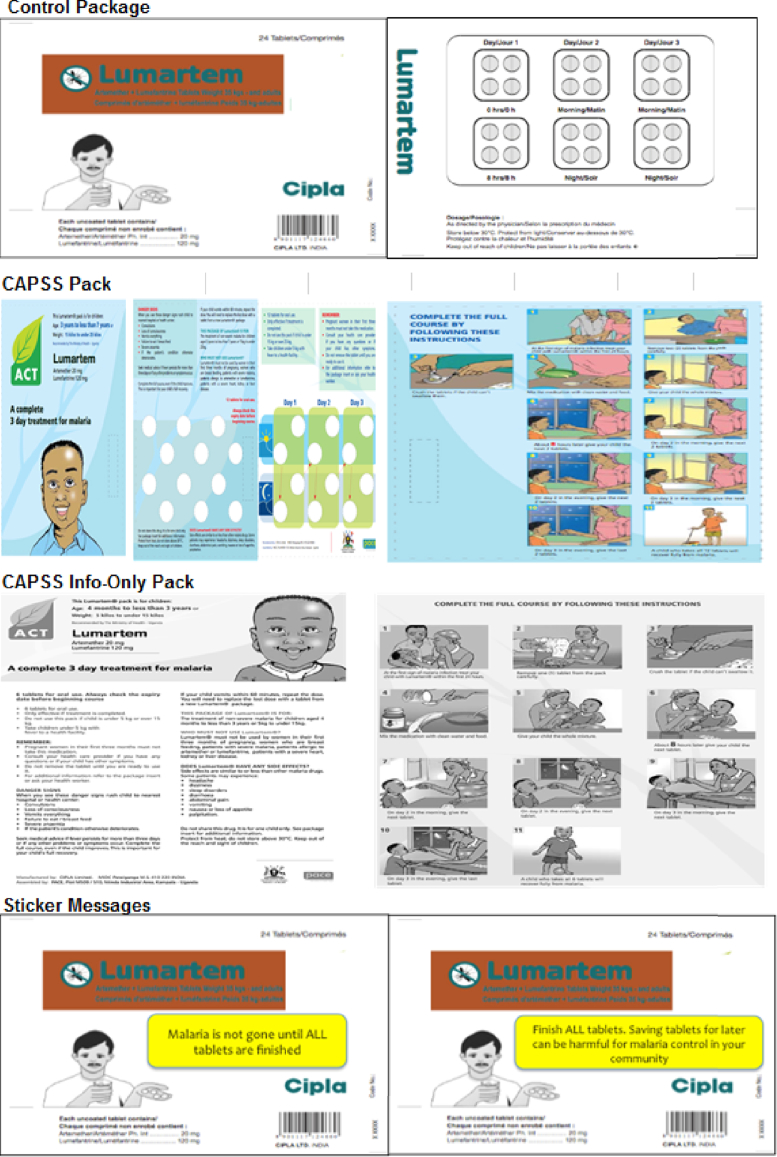
Control and Intervention Packages. The CAPSS pack shown here is for ages 3–7 years. The packages for the other dosage groups are very similar.

Shops were randomized by day into either a control package or one of four treatment packages, shown in Fig. 3. There were two main objectives to the study design. The first was to test the status quo approach to promoting adherence through specialized packaging (the “CAPSS Package”). The second was to test whether some simple, inexpensive additions to the standard ACT package (something that a pharmaceutical manufacturer could easily implement on a large scale) could increase adherence rates. Since Uganda does not have a national language, and because we wanted to test interventions that did not need to be tailored at the national (or sub-national) level, all packages were in English. Though many Ugandans do not speak or read much English, the CAPSS Package – which we did not develop – was already in English. The messages we developed used very simple language with English words that were field tested to be familiar to many Ugandans.

A randomized, cross-cutting intervention was conducted in which a rapid diagnostic test (RDT) for malaria was offered to 25% of patients at the time they purchased ACTs. We evaluate the impact of diagnostic testing on adherence in a separate paper and show that a confirmed diagnosis of malaria had no significant effect on adherence (Saran et al., 2016). For the analysis of the packaging and messaging treatments presented here, we always control for the (orthogonal) RDT offer and present robustness checks showing the impact of the treatment arms for the 75% of patients who were not randomly assigned to be offered testing.

#### 3.2.1 Control package

The control package in this study was the standard package in which Lumartem was sold in Uganda and elsewhere in Africa. The box, shown in Fig. 3, had the name, brand and manufacturer of the medication. Inside the box was a blister pack which grouped the pills by dose and day and a paper insert –similar to what is seen inside most medication boxes in the United States and elsewhere – with small print about dosing, side effects, etc.

#### 3.2.2 CAPSS package and CAPSS Information Only pack

We refer to the first treatment as the “CAPSS” package since it was the ACT package used in Uganda during the Consortium for ACT Private Sector Subsidy pilot program (run by the Uganda Ministry of Health, Medicines for Malaria Venture, Population Services International and others). The CAPSS program was a pilot designed to test the feasibility of a private sector ACT subsidy prior to the Affordable Medicines Facility-malaria (AMFm) – a global ACT subsidiy program that was established in 2009 and implemented in seven African countries, including Uganda (the AMFm program was scaled up in Uganda towards the end of 2011, after this study had already ended) (Gelband et al., 2004; Laxminarayan and Gelband, 2009; Fink et al., 2013).^14^ The ACT CAPSS package, which is similar to packaging used for other ACT subsidy programs in Tanzania and in Rwanda, was intended to serve several purposes. First, it differentiated the subsidized private sector ACTs from those in the public sector (which were intended to be free). Second, it served as a form of branding and quality assurance, providing “consumers with the instant recognition that they were purchasing a high quality and effective anti-malarial at an affordable price” (Talisuna et al., 2012). Finally, it was designed to encourage correct use of the product, incorporating features like colorful pictorial instructions on how to take the medicine, principally to assist illiterate patients and caregivers. Several messages on the CAPSS package relate directly to adherence, such as: 1) “Complete the full course, even if the child improves. This is important for your child's full recovery.”, 2) “Only effective if treatment is completed.”, and 3) “Do not share this drug.” These messages are just a small part of all of the information on the package, including information related to side-effects, storage, proper dosing, etc.

While the potential benefits to this type of specialized packaging are substantial, the CAPSS package, and others like it, add roughly 15–20 cents to the cost of the ACT and can be a source of bottlenecks in the drug supply chain. Because the costs are high, we also tested a packaging type that conveyed the same information content at a significantly lower cost. We created a handout that was a black and white photocopy of the CAPSS package and wrapped it around the control package when distributing the medication at the drug shop. The purpose of this treatment arm was to explore, if the CAPSS package was successful at increasing adherence rates, whether the improvement was due to the information and pictorial instructions, or whether it was also linked to the product quality and differentiation conveyed by the special, glossy packaging. We refer to this second treatment as the CAPSS-Information Only pack.

#### 3.2.3 Simple sticker messages: “Malaria is not gone until…” and “Don't save pills…”

We also tested simple, targeted messages to promote adherence delivered via stickers attached to the control packaging, an approach that is often used to encourage patients to finish their antibiotic treatment. The first sticker said “Malaria is not gone until ALL tablets are finished” and was designed to address non-adherence based on the belief that the illness is cured when symptoms have resolved.^15^ The second sticker said “Finish ALL tablets. Saving tablets for later can be harmful for malaria control in your community.” This message aimed to discourage the saving of pills for the next malaria episode, while encouraging patients to internalize the externality associated with non-adherence. Both stickers were yellow and placed in the front and center of the box of medicines.^16^

### 3.3 Survey tools and measurement

Surveys were conducted at four points through the study period: at baseline, at the drug shop during the time of ACT purchase, several days after ACT purchase (“follow up”), and at study endline. The baseline survey was conducted in the home with the female head of household and collected information about demographics and about malaria treatment and prevention activities. The second point of survey was at the time of ACT purchase, and was administered at the shop with the patient or with the caretaker if the patient was a young child. Among patients aged 12 and above (i.e. patients who were old enough to answer for themselves), 71% of patients were at the shop at the time of the ACT purchase. The questions at the drug shop primarily concerned the severity of the symptoms that the patient was experiencing, and their beliefs about the likelihood that the illness was malaria.

The follow-up surveys took place three days after ACT purchase at the home of patients whose households were ex-ante randomly assigned to receive a follow-up visit. The main purpose of this survey was to determine whether the patient had completed their medications by counting the number of pills remaining in the medication blisterpack. The follow-up survey also included questions about the day and approximate time the patient took each dose of the drug, how sick they felt each day while taking the medication, and their current level of health. The respondent for the follow-up survey was the patient if the patient was 18 years old or above, and the caregiver if the patient was under the age of 12. If the patient was between the ages of 12 and 18, the patient was interviewed in the presence of the caregiver.

At the end of the data collection period, field officers visited each of the participating households and informed them that the study was ending. At this time, field officers collected the Purchase ID Card and asked the female household head a few more questions about their knowledge and beliefs about malaria treatment and elicited their understanding of the dosing instructions on the packages used in this study. The enumerators discussed the benefits of adhering to treatment regimens and informed respondents that a national ACT subsidy program (the AMFm, described above) was now launched in Uganda.

Adherence is defined as having no remaining pills in the blisterpack at the time of the follow-up survey. In the 13 percent of cases where the blisterpack was not seen, we used the patient or caregiver's report on the number of pills remaining. This definition of adherence is standard in the literature, with the majority of studies using a combination of pill counts and self-reports in order to measure adherence (Bruxvoort et al., 2014; Banek et al., 2014). In order to explore whether unseen blister packs were biasing the results, we conducted a robustness check on only the sub-sample who showed the blister pack and we estimated treatment effects under different assumptions about adherence for those who did not show their blister packs. In addition to whether the patient fully adhered, we also look at the number of doses and tablets remaining as additional outcomes of the intervention. Any improvement in the intensive margin is likely to still be beneficial both in treating the disease and in minimizing the likelihood of the development of resistance by reducing the number of parasites remaining in the patient (Stepniewska et al., 2010).

### 3.4 Ethics approval and trial registry

Ethical approval for this study was given by the Harvard T.H. Chan School of Public Health (protocol # CR-19527-02) and the Uganda National Council for Science and Technology (protocol # HS-832). The trial was registered at https://www.socialscienceregistry.org with registry number AEARCTR-0000490. The primary outcomes were pre-specified but the secondary outcomes and heterogeneity analysis were not.

## 4 Results

We begin our discussion of results with a description of the uptake of ACTs sold through the program and some basic characteristics of the sample as well as balance across treatment arms. In Section 4.1 we present basic results on adherence and medication-taking behavior in the sample. We then present visual evidence and regression-adjusted estimates of the impact of the interventions. We drop the 34 ACT purchases where no medication was taken at all (i.e. the entire treatment course was remaining). Assuming the patient had malaria, the parasites were not exposed to the drug, and, therefore, were not under selective pressure to develop resistance to the drug (White et al., 2009). Our main analyses also exclude the 78 patients who were found for the follow-up visit more than 96 h after they purchased the ACTs. In the Appendix, we show that our results are robust to these sample criteria.

Since the randomization of the packages was at the shop-day level, individuals (and households) could have purchased ACTs with different types of packaging over the course of the study period.^17^ Our main model uses all of the ACT purchases (for which we have outcome data) and controls for any previous package types purchased, but in the Appendix we show the robustness of our results to limiting the sample to only the first ACT an individual purchased as well as the robustness to limiting the sample to the first ACT purchased within a household. In order to be conservative, we present estimates with standard errors clustered by shop rather than by shop-day (the level of randomization) (Cameron and Miller, 2015), but since we have only 9 shops we also show p-values based on the wild bootstrap procedure described in Cameron et al. (2008). We run OLS regressions of the following form in our analysis:

(1) $$\begin{array}{cc} y_{isd} & =\beta_{0}+\beta_{1}CAPSS_{sd}+\beta_{2}CAPSS-INFO-ONLY_{sd}\\ & \;+\;\beta_{3}“MALARIA-NOT-GONE”-MESSAGE_{sd}\\ & \;+\;\beta_{4}“DONT-SAVE-PILLS”-MESSAGE_{sd}\\ & \;+\;\sigma_{\mathrm{shop}}+\delta_{\mathrm{day}}+\gamma_{\mathrm{purchase}}+\lambda_{\mathrm{previous}}+RDT+\varepsilon_{isd} \end{array}$$

where *y*_*isd*_ is the outcome for person i who bought an ACT at shop *s* on day *d*. Outcomes include a binary adherence measure equal to one if all medication was completed at the time of follow up and zero otherwise, a “tablets left” variable measuring the number of tablets remaining in the blister pack and a “doses left” variable which is the number of tablets remaining divided by the appropriate number of tablets per dose according to the age of the patient. We control for shop (*σ*_*shop*_) and day (*δ*_*day*_) fixed effects, for the number of times the patient had bought ACTs through the study (including the current purchase)- ie. the ACT purchase number (*γ*_*purchase*_), previous pack types received (*λ*_*previous*_), and a vector of controls for the cross-cutting RDT treatment including a main effect and interactions with the pack type received.

Since the CAPSS and CAPSS-Information Only packages contain additional information from the two sticker messages and also vary substantially in the way the information is presented, we also group together these two types of interventions and estimate a pooled

(2) $$\begin{array}{cc} y_{isd} & =\beta_{0}+\beta_{1}CAPSS/CAPSS-Information-Only_{sd}\\ & \;+\;\beta_{2}STICKER-MESSAGES_{sd}\\ & \;+\;\sigma_{\mathrm{shop}}+\delta_{\mathrm{day}}+\gamma_{\mathrm{purchase}}+\lambda_{\mathrm{previous}}+RDT+\varepsilon_{isd} \end{array}$$

where the CAPSS/CAPSS-Info-Only treatment combines patients who received either the CAPSS package or the CAPSS-Information-Only package, while the Sticker Messages treatment combines patients who received either of the two sticker messages.

### 4.1 Uptake of ACTs, sample characteristics and balance

Over the study period, 42 percent of households (16 percent of individuals) purchased at least one treatment course of ACT using their ID card. The mean number of ACTs purchased per household (individual) was 0.95 (0.20). We do not see much evidence for hoarding: 97 percent of study participants who ever purchased an ACT purchased only one or two courses during the study.

Sample characteristics and balance across treatment arms are shown in Table 1. The female head of household was interviewed roughly 92 percent of the time. On average, among those who reported any education, female household heads had 7.4 years of education and their spouses had about 8.6; 42 percent of them said they could read a letter written in English (Table 1, Panel A). Households in this region are relatively poor: while nearly 80 percent owned a mobile phone, only 17 percent had access to electricity (Table 1, Panel B).

**Table 1:** Baseline summary statistics and balance tests.

	ACT Purchases Randomly Assigned to Follow-Up Survey					
	Mean in Control Group	CAPSS	CAPSS-Information Only	“Malaria is NOT gone until…” Message	“Don't Save Pills…” Message	Obs
	(1)	(2)	(3)	(4)	(5)	(6)
*A. Characteristics of Interviewed Household Head*						
Age (Years)	32.729 (11.192)	−0.347 [0.801]	−0.295 [0.848]	−0.557 [1.133]	0.125 [1.281]	1695
Female	0.918 (0.274)	0.007 [0.030]	−0.013 [0.020]	0.013 [0.025]	−0.021 [0.020]	1702
Reads English	0.420 (0.494)	0.020 [0.044]	−0.058 [0.043]	−0.025 [0.061]	0.051 [0.040]	1695
Years of Education (Among Those Who Reported Some Education)	7.362 (2.929)	−0.094 [0.255]	−0.293 [0.197]	−0.208 [0.346]	0.259 [0.228]	1570
Years of Spouse/Partner Education (Among Those with Some Education)	8.603 (3.124)	−0.262 [0.271]	−0.219 [0.212]	−0.028 [0.401]	−0.581∗ [0.266]	1255
*B. Household Characteristics*						
Household Size	6.012 (2.737)	−0.066 [0.260]	0.284 [0.173]	0.286 [0.213]	−0.004 [0.187]	1702
Has Electricity	0.170 (0.376)	−0.017 [0.031]	0.003 [0.045]	−0.016 [0.038]	−0.014 [0.034]	1689
Owns Mobile Phone	0.790 (0.408)	0.004 [0.026]	0.019 [0.028]	0.018 [0.049]	−0.099∗ [0.050]	1691
*C. Health Behaviors and Knowledge*						
Member of Household had Malaria in the last 30 days	0.745 (0.436)	−0.070 [0.060]	0.000 [0.051]	−0.066∗ [0.030]	−0.072 [0.060]	1702
Slept under Bednet Last Night	0.640 (0.480)	0.032 [0.045]	−0.006 [0.047]	0.034 [0.057]	0.014 [0.043]	1611
Heard of ACTs	0.663 (0.473)	−0.021 [0.029]	−0.029 [0.048]	−0.124∗∗ [0.052]	−0.007 [0.047]	1702
*D. Treatment-Seeking Behavior for Previous Malaria Episode*						
Sought Treatment at Drug Shop	0.304 (0.461)	0.056 [0.066]	0.022 [0.079]	0.044 [0.062]	−0.044 [0.046]	612
Sought Treatment at Private Hospital Or Clinic	0.429 (0.496)	−0.132 [0.077]	−0.085 [0.104]	−0.055 [0.050]	−0.061 [0.081]	612
Received Confirmed Diagnosis (Microscopy or RDT)	0.153 (0.361)	0.106 [0.084]	0.006 [0.065]	−0.005 [0.052]	0.031 [0.043]	609
Used ACT (Among Those Taking Medicine)	0.527 (0.501)	−0.095 [0.074]	−0.060 [0.054]	−0.019 [0.081]	−0.103 [0.068]	472
*E. ACT Purchases During Study*						
Number of ACT Purchases per Individual	1.430 (0.652)	0.001 [0.059]	0.017 [0.048]	−0.060 [0.049]	0.018 [0.037]	1702
Age (Years)	14.432 (17.004)	−1.370 [1.274]	−0.569 [1.389]	−2.676∗∗ [1.076]	0.634 [1.006]	1682
% of Adult Dose Purchases (Aged 12 years and above)	0.378 (0.485)	−0.001 [0.043]	0.012 [0.053]	−0.032 [0.046]	0.039 [0.037]	1696
Offered Free Rapid Diagnostic Test (RDT) for Malaria^a^	0.245 (0.430)	0.016 [0.031]	0.003 [0.030]	−0.007 [0.033]	−0.062∗ [0.030]	1702
Tested Positive for Malaria on RDT^a^	0.655 (0.478)	−0.014 [0.133]	−0.042 [0.093]	−0.159∗ [0.082]	−0.102 [0.120]	361

Column 1 shows the mean and standard deviation (in parentheses) in the control group of the ‘analysis sample’: patients who purchased ACTs who were followed up within 96 h and who started taking their medication. Columns 2–5 show the coefficients on dummies for each of the treatment groups with standard errors (clustered by shop) in square brackets. Regression controls are presented in Equation (1). ^∗^p < 0.10, ^∗∗^p < 0.05, ^∗∗∗^p < 0.01.

a Does not include controls for whether RDT was offered and interactions between each pack type and the RDT offer.

Roughly 75 percent of households reported having a member with suspected malaria in the month prior to the baseline survey and about 64 percent of household members slept under a mosquito net the night before the survey. At baseline, 66 percent of female household heads had heard of ACTs (Table 1, Panel C). Among patients who sought outside treatment for a previous episode of malaria (almost everyone did), 30 percent first sought care at a drug shop, while 43 percent first sought care at a private hospital or clinic (the remaining 26 percent visited a public health center or hospital). Only 15 percent received a confirmed diagnosis of malaria using microscopy or an RDT and roughly 53 percent of those who took medicines to treat the illness took ACTs (Table 1, Panel D).

Just over 60 percent of ACT purchases during the study were for children in the three lower age/dose categories (under 12 years old), while the remaining 38 percent were for the highest dosage category (individuals ages 12 and older). A small subsample (25%) of patients was randomly tested for malaria at the time of ACT purchase. Positivity rates were 66 percent overall for this subsample (higher for children specifically) and though there are some differences in positivity rates across treatment arms, these are likely due to the very small sample size (N = 361) split across five arms (Table 1, Panel E).

While there are some statistically significant differences in the characteristics between treatment arms and the control group, for most of the arms, only one or two variables are statistically significant, the differences are all modest in magnitude, and they don't seem to vary systematically with treatment arm. The “Malaria is not gone until…” treatment group has a few notable differences from the control group, but these differences do not suggest any particular pattern. Households in this arm reported less malaria in their household, which would suggest that they were somewhat older and of higher socioeconomic status than households in the control group. However, households in this arm are actually somewhat younger on average than those in the control and do not appear to differ from the control in any measure of socioeconomic status. We present analyses in the appendix that test the robustness of our main results to including controls for these variables in our regression.

Appendix Table A2 shows loss-to-follow-up across treatment arms. 96% of follow-ups were completed, with attrition balanced across treatment arms other than the “Don't Save Pills…” group, which was 4.4 percentage points less likely to have a completed follow-up visit than the control group. Those who received the CAPSS pack were approximately 7 percentage points less likely to have their blisterpack available at the followup visit.^18^^,^^19^

### 4.2 Overall adherence behavior

The overall adherence rate in the control group was 63.8 percent, with a mean number of doses left of 0.84 (Table 2). Non-adherent patients in the control group had an average of 2.3 doses (just over a day's worth) left (results not shown). Adherence was high for the first two doses (95 percent and 90 percent) and then fell steadily (between 8 and 11 percent percentage points) with each subsequent dose (results not shown).

**Table 2:** Impact of packaging interventions on ACT adherence.

Coefficient on:	By pack type			Pooled		
	Adhere	Doses	Tablets	Adhere	Doses	Tablets
	(1)	(2)	(3)	(4)	(5)	(6)
A. CAPSS	−0.031 (0.038) [0.433] {0.472}	0.022 (0.106) [0.837] {0.832}	0.162 (0.359) [0.663] {0.642}			
B. CAPSS-Info Only	−0.033 (0.034) [0.349] {0.370}	0.183 (0.147) [0.250] {0.244}	0.539 (0.529) [0.338] {0.352}			
C. “Malaria is not gone until…” Sticker Message	0.059∗∗∗ (0.014) [0.004] {0.000}	−0.231∗∗ (0.093) [0.038] {0.012}	−0.704∗∗ (0.300) [0.047] {0.020}			
D. “Don't save pills…” Sticker Message	0.055 (0.048) [0.286] {0.326}	−0.160 (0.101) [0.152] {0.152}	−0.600 (0.387) [0.159] {0.194}			
E. CAPSS/CAPSS-Info Only (A and B combined)				−0.027 (0.026) [0.329] {0.280}	0.091 (0.106) [0.416] {0.392}	0.318 (0.386) [0.433] {0.386}
F. Sticker Messages (C and D combined)				0.057∗ (0.030) [0.094] {0.080}	−0.194∗∗ (0.073) [0.029] {0.056}	−0.647∗∗ (0.273) [0.045] {0.050}

Mean of Outcome in Control Group	0.638	0.839	2.261	0.638	0.839	2.261
P value: (C = D)	0.918	0.601	0.815			
P value (E = F)				0.002	0.002	0.002
R squared	0.139	0.142	0.193	0.132	0.135	0.189
Number of Obs	1702	1698	1698	1702	1698	1698

Specification for Columns 1–3 in Equation (1); specification for Columns 4–6 in Equation (2). Regressions with tablets as an outcome also include dosage group fixed effects. Sample is limited to those who started taking the medication and who were visited for a follow-up survey within 96 h of ACT purchase. Standard errors are in parentheses and clustered at the shop level, p-values in square brackets. P-values using wild bootstrap clustered standard errors are in curly braces. ^∗^p < 0.10, ^∗∗^p < 0.05, ^∗∗∗^p < 0.01.

### 4.3 Impact of packaging and messaging on adherence

#### 4.3.1 Graphical evidence

**Fig. 4:**
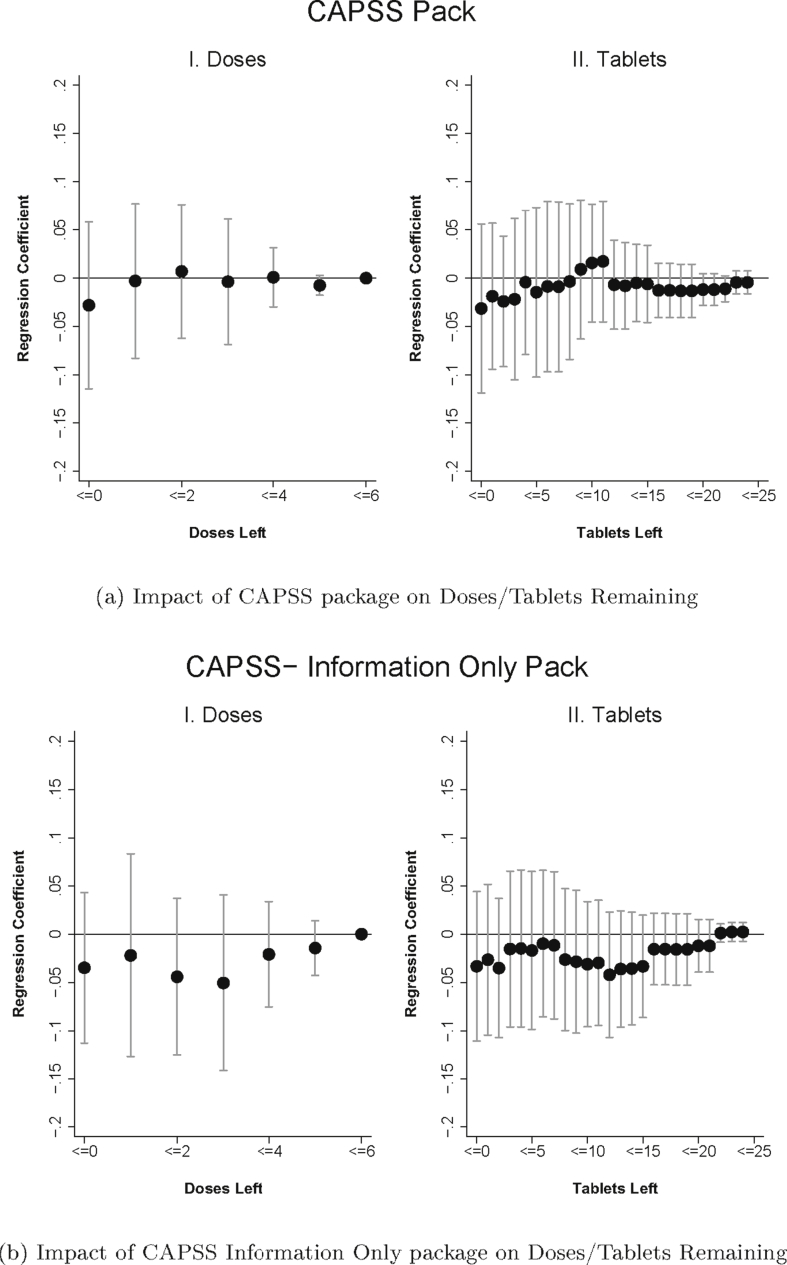

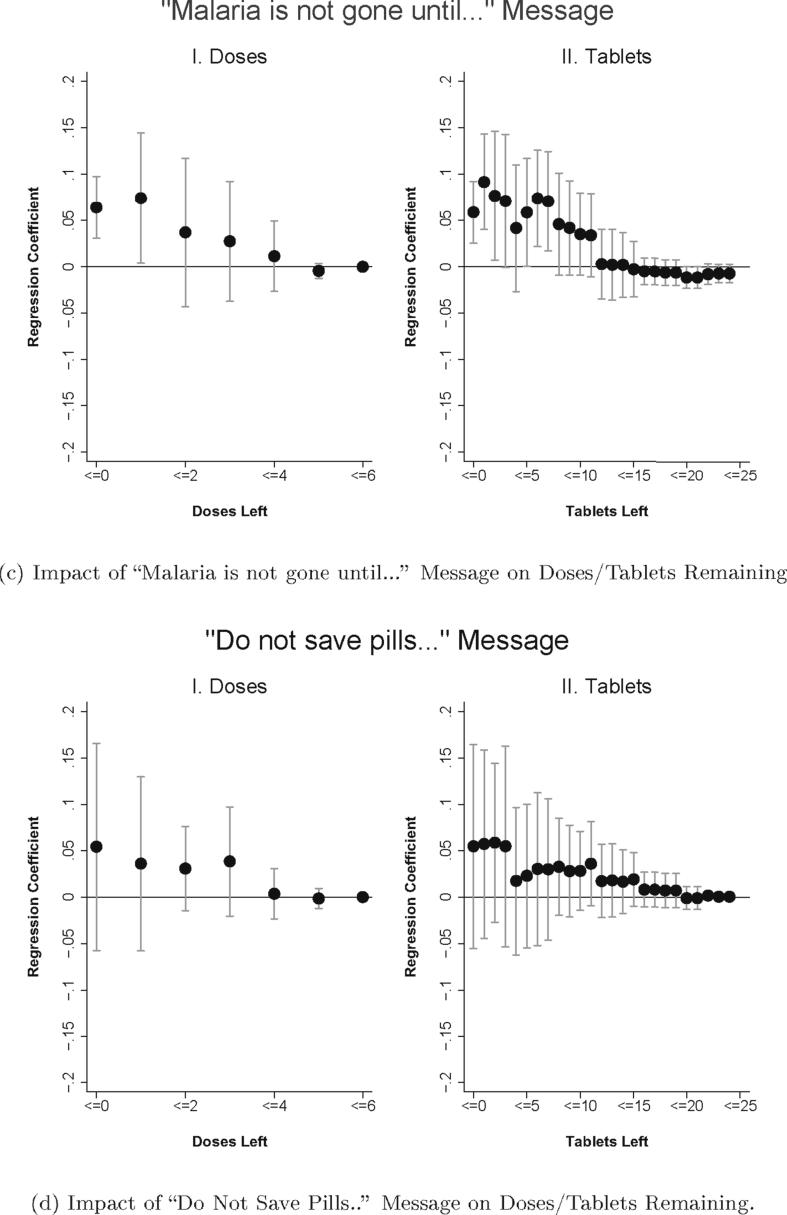
Figures plot regression coefficients of the impact of the treatment (compared to the control) on the cumulative probability of each dose (Panel I) or of each tablet (Panel II) remaining with 95% confidence intervals. The regressions include the controls specified in Equation (1). Regression with tablets remaining also include dosage fixed effects. Sample is limited to patients who were followed up within 96 h of ACT purchase and who started taking the medication. Standard errors are clustered at the shop level.

We start by presenting a simple graphical analysis of the impact of packaging on adherence (Fig. 4a–d). For each treatment arm, we present two figures. The figure on the left plots the treatment coefficients (and 95 percent confidence intervals) from a regression in the form of Equation (1), but with a series of dummy variables for outcomes indicating “zero doses left” (i.e. full adherence), “one or fewer doses left”, “two or fewer doses left”, etc. The figure on the right shows the coefficient on tablets remaining instead. Graphical evidence of a positive treatment impact would be seen in the coefficients for a treatment arm lying above zero.

Fig. 4a shows the impact of CAPSS packaging relative to the control for doses and tablets remaining. The figure suggests that there is no impact of CAPSS on medication taking, as the coefficients are close to zero, although the confidence intervals are very wide. We also do not see evidence that the “CAPSS Information Only” arm increases adherence (Fig. 4b). The point estimates are negative though quite noisy. Taken together, these results suggest that the current approach to promoting adherence through specialized packaging is not effective at improving adherence.

The impact of the “Malaria is not gone until…” message on adherence is presented in Fig. 4c. While the difference in the probability of having five or fewer doses remaining is not affected by the message, the impact increases in magnitude and statistical significance as doses left decrease (and as tablets left decrease, see Fig. 4c, Panel II), suggesting that the message leads to improvements in medication taking at the later stages of the treatment course. Fig. 4d shows the impact of the “Don't Save Pills” message on adherence. Although the point estimates on adherence by dose and tablet are positive–with a similar pattern of increasing impact as doses/tablets decline–the confidence intervals are wide and include a range of impact estimates.

#### 4.3.2 Regression estimates and robustness

Regression estimates based on Equation (1) and Equation (2) are presented in Table 2. Column (1) presents coefficient estimates of the impact of each treatment arm on adherence. As seen in the figures, the CAPSS and CAPSS-Information Only arms have insignificantly negative impacts on adherence, while the “Malaria is not gone until…” message and “Don't save pills…” messages have positive effects on adherence that are very similar in magnitude, though only the “Malaria is not gone until…” message is statistically significant. The “Malaria is not gone until…” message increases adherence by 5.9 percentage points (9.2 percent), relative to the mean of 63.8 percent adherence in the control group. While the effect on overall adherence is modest, the magnitude of its effect on the number of pills remaining is more substantial. The “Malaria is not gone until…” sticker reduces the number of doses remaining by 0.23, a 27 percent decrease in remaining doses (Column 2), and reduces the number of tablets remaining by 0.70, a 31 percent reduction in remaining tablets (Column 3). The coefficient estimates on the “Don't save pills…” message are similar to the other sticker for all outcomes, but are not statistically significant.

In Table 2 Columns 4–6 we show the pooled estimates from Equation (2). The sticker interventions increase adherence by 5.7 percentage points (8.9 percent) while the CAPSS/CAPSS Info Only packages reduce adherence by a statistically insignificant 2.7 percentage points. The sticker interventions decrease the number of doses left by roughly 0.2 (23 percent) and number of pills left by 0.65 (29 percent). An F-test confirms that the effects of these two types of messages are statistically different (p = 0.002).

Appendix Table A3 presents several robustness checks. Columns 1 and 2 limit the sample to the first ACT purchased by an individual and the first ACT purchased by a household, respectively. We also test the robustness of our estimates to our sample definition by including patients who were visited for a follow-up survey after 96 h (Column 3) and by including patients who did not start taking their medication (Column 4). In Column 5, we limit the sample to those who showed their blisterpack at the follow-up visit and in Column 6 we control for variables that were imbalanced at baseline. We find similar impacts as in the main analysis. The “Malaria is not gone until…” message increases adherence rates by 4.1–7.8 percentage points and is generally statistically significant, while the coefficients on the “Don't Save Pills…” message are always positive and similar to the other sticker, ranging from 4.6 to 8 percentage points, but not statistically significant. The CAPSS and CAPSS-Info Only packages seem to reduce adherence rates but the coefficients are not generally statistically significant. In Appendix Table A4, we examine the robustness of our results to limiting the sample to those who were not offered a free rapid diagnostic test for malaria. The results are similar to the main results in Table 2, albeit somewhat less precise.

Appendix Table A5 displays the robustness of our main results to three different assumptions about adherence rates among those who did not have their medication blisterpack available at the time of the follow-up survey. We assumed that everyone who did not show the blisterpack either all finished their medication (Column 1), or all did not finish the medication (Column 2), or that the adherence rates among those who did not show their blisterpack was the same as those who did show their blisterpack, separately by the type of package that they received (Column 3). As in our main results, the “Malaria is not gone until…” message increases adherence rates by 5.3–5.8 percentage points and is statistically significant. Under the assumptions that those who did not show their blisterpack did not adhere, the CAPSS package actually reduces adherence rates by 10.9 percentage points.

## 5 Patterns of ACT adherence and Heterogeneous effects

In this section we explore how adherence varies with some of the factors highlighted in the theoretical framework in Section 2 and explore whether the impact of the interventions varied with these characteristics.

### 5.1 Adherence in the control group

**Fig. A3:**
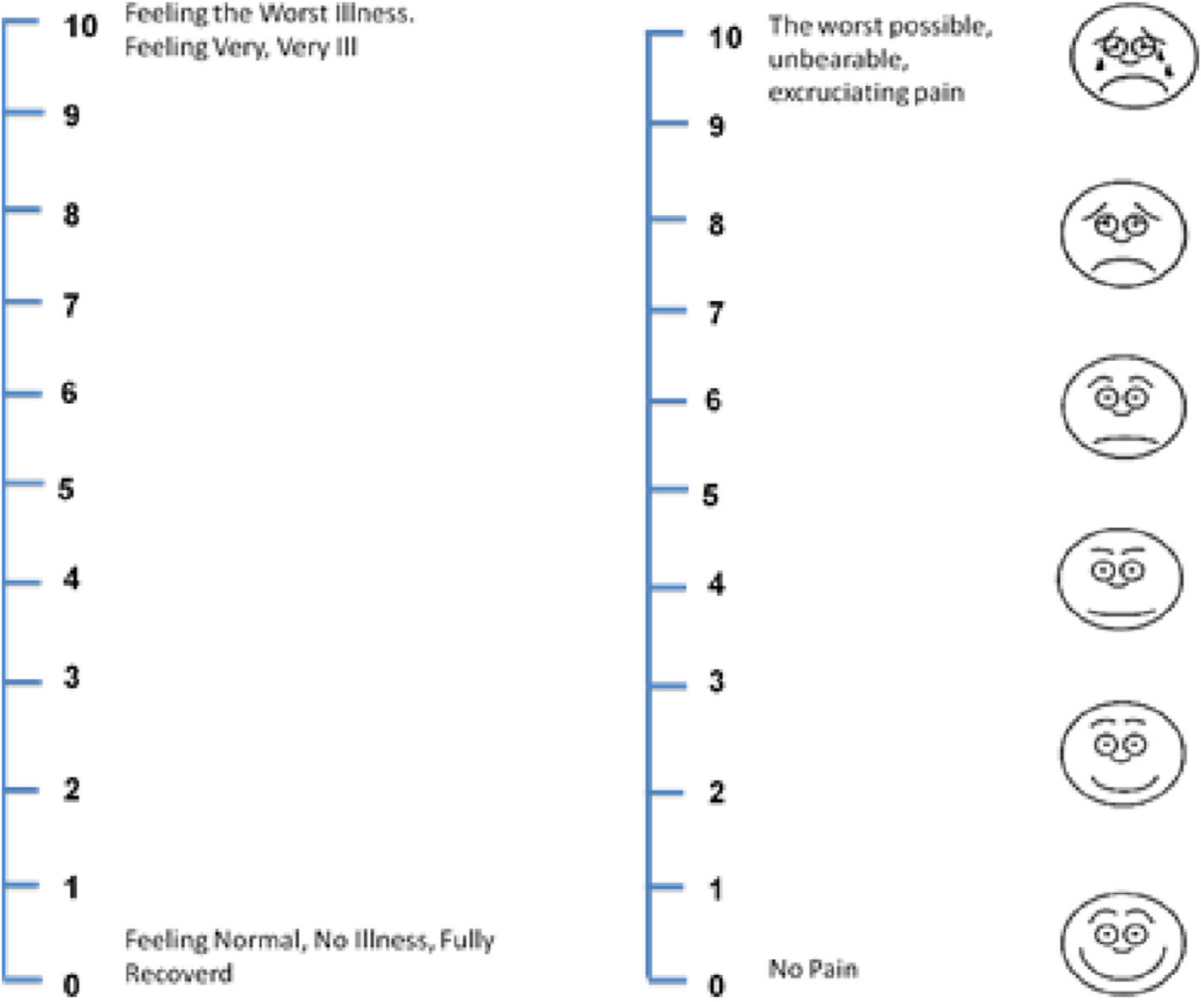
Ladder scales used to gauge severity of symptoms.

Our theoretical framework highlights the potential importance of mid-treatment symptom severity, beliefs about when the illness is cured, and beliefs about the effectiveness of the drugs in a patient's adherence decision. In the follow-up survey, individuals were shown a 0–10 ladder scale (a visual analog scale) and asked to indicate how they felt on each of the three to four days they were taking the medication. The top of the scale (10) indicated the “worst feeling of illness”, while the bottom of the scale (0) implied that they felt in perfect health (see Appendix Fig. A3). For those who no longer believed they had malaria, we asked them which day they believed their malaria went away (with Day 1 being the first day of treatment).

**Fig. 5:**
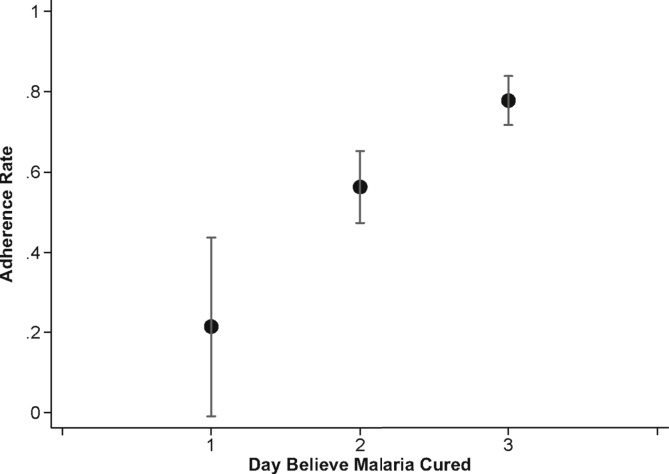
Adherence in the Control Group by Day Believed Cured. Mean adherence rates for individuals who received the control package by the day they believed they were cured. Day 1 is the day that they started taking treatment. Sample is limited to people who believed their malaria went away, who started taking the medication, and who were visited for a follow-up survey within 96 h of ACT purchase. In addition, adherence for the 5 people (1.57% of all respondents) who reported being cured on the fourth day of treatment is not shown due to small sample size.

**Fig. 6:**
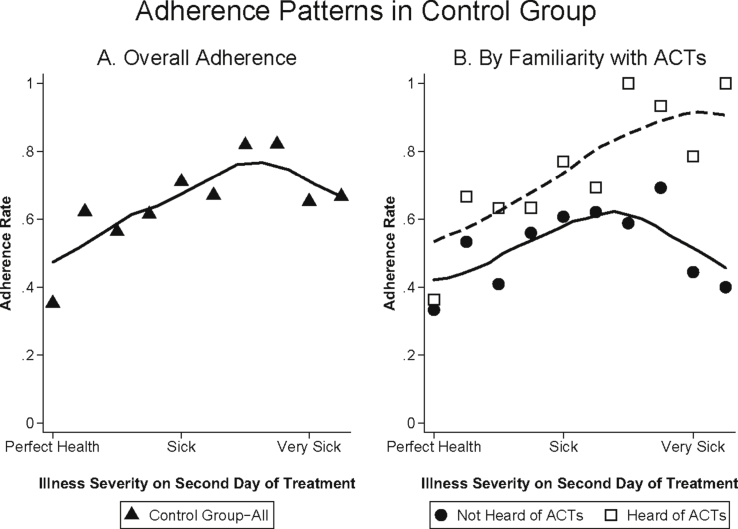
Adherence Patterns in the Control Group by Symptom Severity. Mean adherence rates for individuals by symptom severity on the second day of treatment for all those who received the control package (Panel A) and separately by whether or not the household respondent had heard of ACTs prior to the baseline survey (Panel B). Symptom severity was measured on a 10-point scale with larger numbers indicating increasing levels of sickness. Sample is limited to people who started taking the medication and who were visited for a follow-up survey within 96 h of ACT purchase. Adherence for the 3 individuals who reported an illness severity of 10 (0.6% of all respondents), is not shown due to small sample size and because this was only reported for individuals in the group that had heard of ACTs. The sample sizes in Panel A for illness severities 0–9 are as follows: 17,45,71,96,76,91,39,28,23,9. The sample sizes in Panel B for illness severities 0–9 are as follows: 6,15,22,25,28,29,17,13,9,5 (for those who hadn't heard of ACTs) and 11,30,49,71,48,62,22,15,14,4 (for those who had heard of ACTs).

**Fig. A4:**
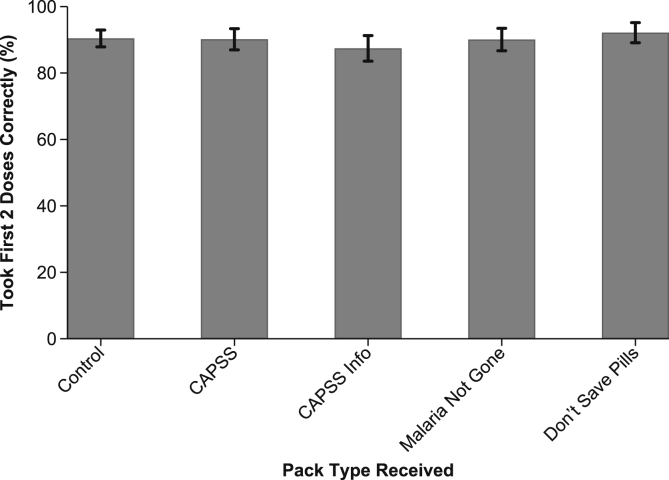
Timely adherence for First 2 Doses. We use the time that people took their first dose to construct a variable for whether each subsequent dose was taken at the correct time, and using the correct number of pills. Timings are approximate (morning, afternoon, evening). Sample is limited to those who started taking the medication.

Fig. 5 plots adherence rates by the day individuals said they believed their malaria went away. In the control group, people who believed they were cured on Day 1 were 56 percentage points less likely to adhere than those who believed they were cured on Day 3 (P < 0.001). Fig. 6 plots the relationship between adherence and symptom severity on the second day of treatment for those who received the standard control pack. While symptoms on day two are, of course, partly themselves a function of adherence, nearly everyone adhered on the first day of treatment (See Appendix Fig. A4). Fig. 6A demonstrates that adherence is strongly related to mid-course symptom severity, with the probability of adherence nearly 15 percentage points higher (P < 0.001) for those who felt moderately sick, and approximately 5 percentage points higher (P = 0.458) for those who felt very sick, compared to those who felt well on the second day of treatment (these categories of illness severity correspond, on the 10-point scale, to illness levels 0–3 for “well”, 4–7 for “moderately sick” and 8–10 for “very sick”).

48% of households (31% of ACT purchasers) reported that they had never heard of ACTs at baseline. Fig. 6B demonstrates the relationship between adherence and mid-course symptom severity separately for those who were and were not already familiar with ACTs. Adherence among those who had not previously heard of ACTs (solid line) follows the predicted inverse U-shaped pattern from the theoretical framework: adherence for those who still felt moderately sick on the second day of treatment was approximately 14 percentage points higher than adherence for people who felt that they were well (P = 0.037) and 19 percentage points higher than adherence for people who still felt very sick (P = 0.167). On the other hand, there is no drop in adherence at the highest levels of mid-course symptom severity among those who had heard of ACTs at baseline. At the highest symptom severities (levels 8–10), adherence is 33 percentage points (P = 0.102) higher for those who had heard of ACTs compared to those who had not heard of ACTs.^20^

**Fig. A5:**
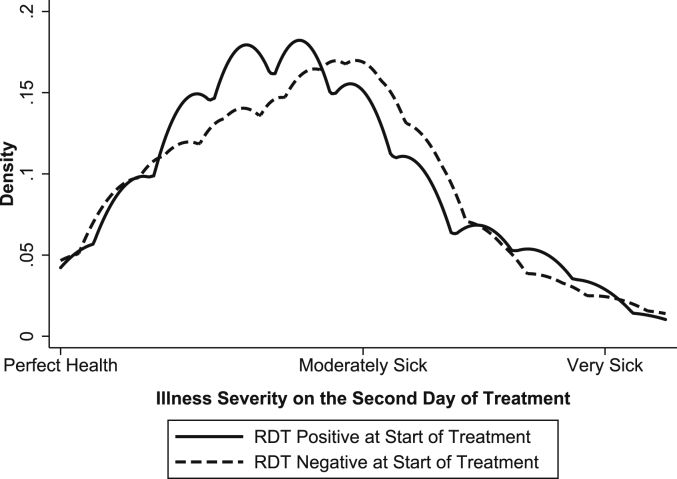
Distribution of Illness Severity on the Second Day of Treatment by Malaria Positivity at the Start of Treatment. The distribution of reported illness severities (using kernel density estimation) on the second day of treatment by whether they tested positive for malaria on the RDT at time of ACT purchase (solid line) or tested negative for malaria on the RDT (dashed line). Sample is limited to those who started taking the treatment.

Overall, our data point toward a very strong relationship between symptom resolution, beliefs about being cured, and ultimate adherence behavior, though of course these are simply correlations. We cannot say with certainty whether patients whose symptoms resolve sooner are more likely to actually be cured of malaria prior to finishing treatment, but we show in Appendix Fig. A5 that patients with earlier symptom resolution are not more likely to have been malaria-negative to begin with.^21^

### 5.2 Heterogeneous effects by sympom severity and beliefs about cure

**Fig. 7:**
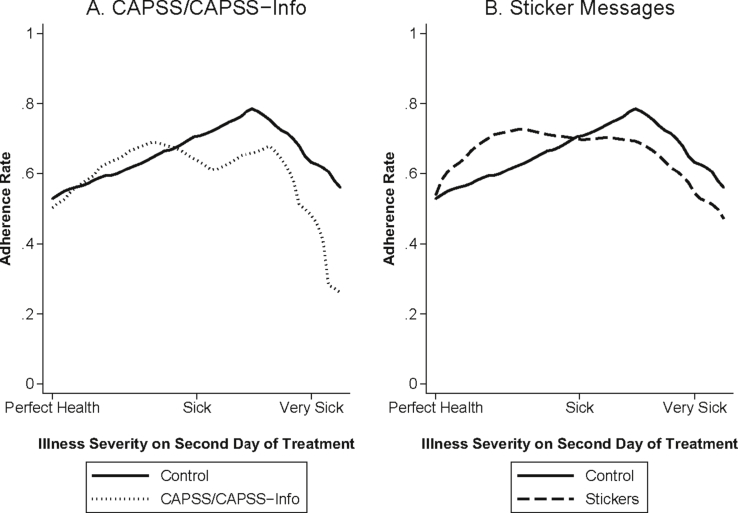
Mid-Treatment Symptom Severity and Adherence. Smoothed local polynomial kernel weight regression of adherence on symptom severity on the second day of treatment. Symptom severity was measured on a 10-point scale with larger numbers indicating increasing levels of sickness. Sample is limited to individuals who started taking the medication and who were visited for a follow-up survey within 96 h of ACT purchase.

**Fig. 8:**
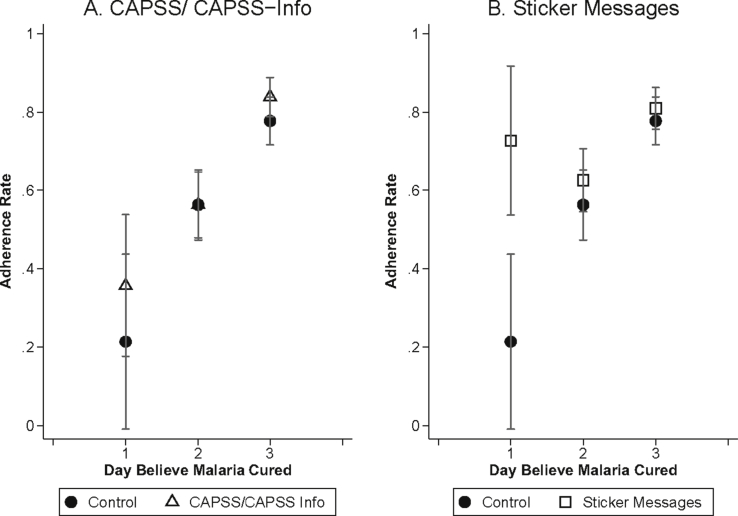
Beliefs about Day Cured and Adherence. Adherence rates (and 95% confidence intervals) according to when patients said, at the follow-up survey, they believed their malaria went away (Day 1 is the day they started treatment). Sample is limited to those who started taking the medication and who were visited for a follow-up survey within 96 h of ACT purchase.

In Fig. 7 we examine heterogenous effects of the interventions by mid-course symptom severity. We plot a local polynomial regression of adherence on mid-course symptom severity, separately for those who received either the control package (solid line), the CAPSS or CAPSS-Information only packages (Panel A, dotted line), or one of the sticker messages (Panel B, dashed line). This figure demonstrates that the stickers were primarily effective at increasing adherence among those who reported feeling much better, increasing adherence by 11 percentage points (P = 0.005) among people who reported symptom levels between 0 and 3 on the 10-point scale. Fig. 8 demonstrates that sticker messages increased adherence among those who believed they were cured on Day 1 by 51 percentage points (P = 0.003), essentially eliminating the association between patient adherence and beliefs about the day when they were cured.

Since the two sticker messages contained different information and targeted specific beliefs about adherence, they could have increased adherence for different reasons. Our main results show that the two stickers had a similar impact on adherence. This suggests that either the stickers corrected misperceptions about adherence, but did so to a similar extent, or that the content of the stickers didn't matter and that they simply made the importance of adherence more salient. While we don't have direct measures of belief updating, our endline survey captured some measures of beliefs about adherence. In Appendix Table A6, we explore whether the type of packaging received during the study influenced endline beliefs. We grouped responses from an open-ended question about what happens if a patient does not finish all their medication into responses that were related to the “Malaria is not gone until…” message (e.g. the patient would feel sicker, would not get better, etc) and those that were related to the “Do not save pills…” message (e.g. members of the household or community would be more likely to get malaria, it would be harmful for the community, etc.). We also include a binary variable indicating whether the respondent “agreed” or “strongly agreed” with the statement “The number of pills of my malaria treatment I should take depends on how sick I feel,” which is closely related to the message contained in the “Malaria is not gone until…message.” The last outcome is a binary variable that indicates whether the respondent “agreed” or “strongly agreed” with the statement “When taking Lumartem to treat malaria, it is important to take the treatment for 3 days.” We did not expect that respondents' beliefs about this statement should vary by the type of sticker message received. We find no significant effects of any of the package types on endline beliefs, other than the CAPSS package increasing beliefs about the importance of adherence for one of the outcomes. It is possible that the CAPSS package did influence beliefs but that this did not translate into increases in adherence, but Table A6 also tests 16 hypotheses (4 outcomes x 4 package types) so this is also possibly just a chance finding. Regardless, these results suggest that the pathway by which the stickers influenced adherence was unlikely to be through belief updating.

### 5.3 Perceptions of drug effectiveness

**Fig. 9:**
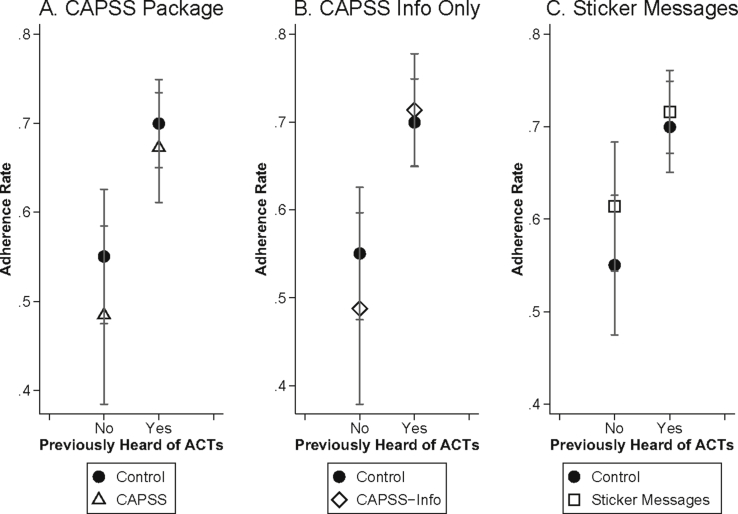
Previous Knowledge of ACTs and Adherence. Adherence rates (and 95% confidence intervals) according to whether individuals had heard of ACTs prior to the baseline survey. Sample is limited to those who started taking the medication and who were visited for a follow-up survey within 96 h of ACT purchase.

A key feature of the CAPSS package is the glossy, colorful packaging, which is intended to convey that the drugs are of high quality and are effective in treating the disease. This may be particularly important for patients who are unfamiliar with ACTs or with their effectiveness. The CAPSS package, however, did not differentially improve adherence for patients who had not heard of ACTs prior to the study (Fig. 9).

### 5.4 Understanding dosing instructions

Another important characteristic of the CAPSS and the CAPSS-Information packs was that they both included pictorial instructions and visual cues to demarcate dosing (see Fig. 3), which are designed to increase patients' understanding of how to correctly take the drugs, particularly for illiterate patients or caregivers. However, we find no evidence that the CAPSS and CAPSS-Information packages significantly increased adherence rates among those who could not read English (Appendix Table A7) and our data suggests that this may be because the CAPSS package does not significantly increase people's understanding of dosing instructions compared to the standard ACT control package (Appendix Table A8). In addition, knowledge of dosing instructions does not appear to be the primary barrier to adherence in this context, since 90 percent of patients (across all pack types) took the first two doses, with the correct number of pills per dose, at approximately the correct time (see Appendix Fig. A4).^22^

## 6 Cost-effectiveness of sticker interventions

In this section, we estimate the cost-effectiveness of the sticker interventions using published malaria cure rates from clinical trials of patients assigned to take 4 doses or 6 doses of AL, the ACT used in this study. We use cure rates from the published literature rather than endline malaria incidence or prevalence rates because, in a context of very high malaria endemicity, extremely large sample sizes would have been required in order to have sufficient statistical power to detect differences in these outcomes by treatment arm.

The additional cost of adding a sticker, such as the ones we used in this intervention, is approximately $0.015 per package. This includes the cost of the sticker itself which is approximately $0.013 and the cost of printing the message on the sticker which is $0.002 (assuming that printing a single page, which consists of 30 stickers, costs $0.06).

**Fig. A6:**
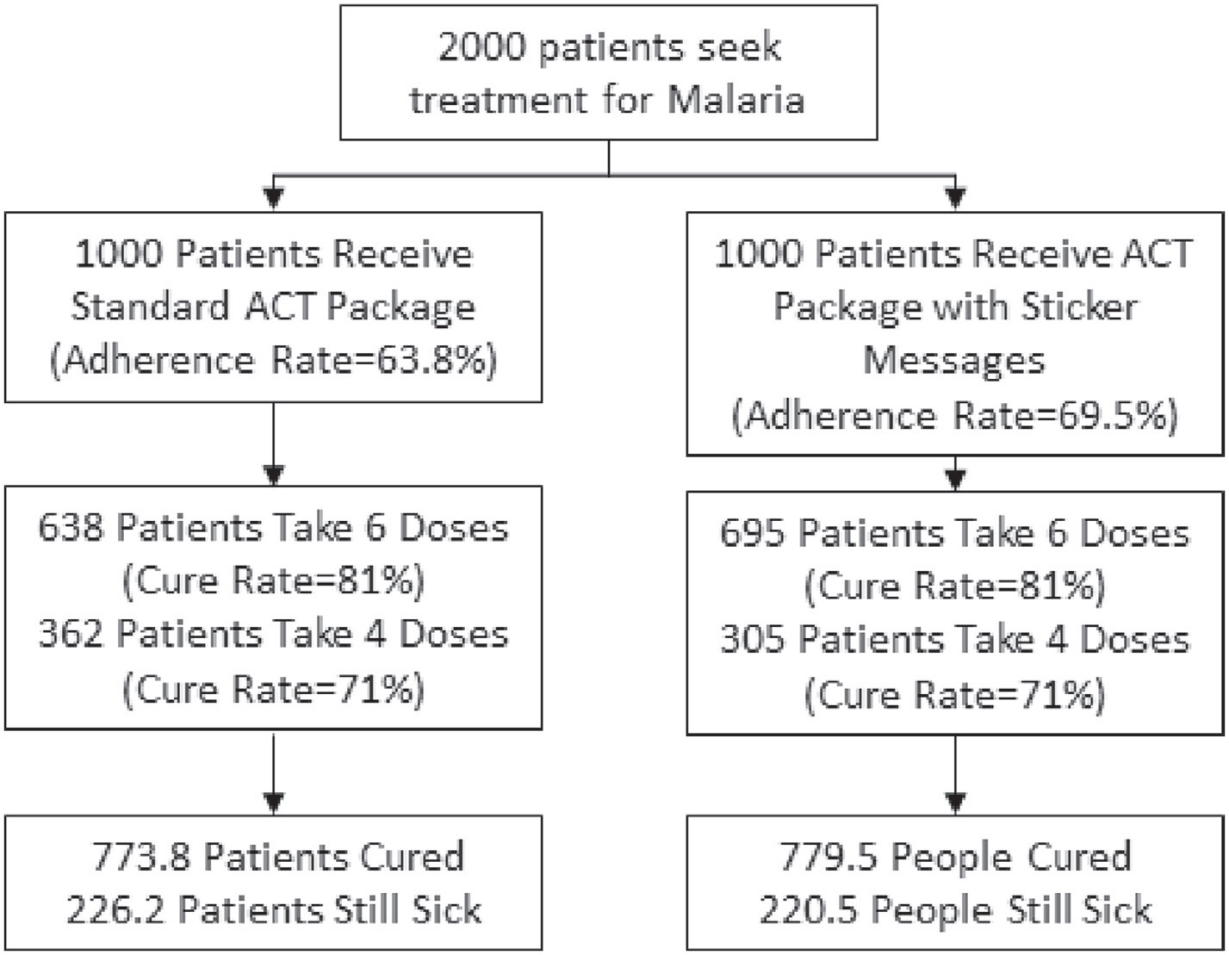
Estimates of number and cost of averted infections with the sticker interventions. We assume that patients who receive the targeted messages on the stickers have an adherence rate of 69.5% while patients who receive the standard ACT package have an adherence rate of 63.8% (see Table 2, Column 4). Patients who do not adhere are assumed to take 4 doses of the medication instead of the recommended 6 doses. Cure rates are from Vugt et al. (1999) and are not PCR-corrected to distinguish new from recurrent infection.

Appendix Fig. A6 outlines the method used to calculate the number of averted infections using the targeted sticker messages. We assume that patients who do not finish the medication take four doses of the drug instead of the recommended six doses. This assumption seems conservative since we find that patients who did not finish the medication had, on average, 2.2 doses remaining at the time of the follow-up visit. In our main specification (Table 2, Column 4), the stickers increased adherence rates by 5.7 percentage points compared to the control group (which had an adherence rate of 63.8 percent). If we assume that everyone who buys the medication actually has malaria, and use cure rates comparing four doses of AL to six doses of AL from Vugt et al. (1999), this results in 5.7 averted infections per 1000 patients receiving the intervention, at a cost of $15. This implies that the cost of a single averted infection using this intervention is approximately $2.63. If we use alternative assumptions about the malaria positivity rates among the sample of ACT-buyers (for example in our sub-sample that was tested, 67 percent tested positive for malaria (74 percent among children under age five)) and about the differential cure rates for four versus six doses of ACTs (Makanga et al., 2006), we get costs per averted infection that range from $0.82 to $3.93 (Appendix Table A9).

## 7 Conclusion

The focus of most interventions to improve medication adherence is on chronic, long-term treatments (McDonald et al., 2002; Haynes et al., 2008). However, sub-optimal adherence to short-course therapies such as antimalarial drugs and antibiotics not only makes it less likely that the disease is cured, but also increases the risk of pathogen resistance to the treatment. We find that 35% of patients do not finish a full course of artemisinin combination therapies for malaria, despite the fact that these are only a three day treatment course. Consistent with previous literature (Banek et al., 2014), we find no relationship between adherence and the age of the patient (data not shown), which is somewhat surprising given that infants and young children are at much higher risk of serious consequences of sub-therapeutic malaria treatment than adults, and because the total number of pills that adults must take is much larger. Currently, in many African countries, the only large scale, patient-focused attempt to increase adherence to over-the-counter ACTs is to add pictorial instructions to enhance comprehension of dosing guidelines. Typically used in branded, “social marketing” campaigns distributing ACTs, the packaging is also glossy and colorful to convey the high quality of the drugs. We find that this common approach is not effective in increasing adherence. This is of particular importance because this type of package adds substantially to the cost of the drugs.^23^ However simple stickers on the standard box of ACTs, with messages that emphasize the importance of completing the medication for curing the disease, are moderately successful in increasing adherence rates.

While the impact of the sticker messages on adherence may not be large enough to affect the probability of parasite resistance, this small addition to ACT packaging has a number of benefits. First, it has a more substantial impact on the number of doses taken which increases the probability of parasite clearance (and hence illness resolution for the patient). Further, adding a sticker with a message on the box of medications is very inexpensive, costing approximately 1.5 cents per package. We estimate that the messages cost between $0.82-$3.93 per averted malaria infection. Thus, these types of stickers are likely to be a very cost-effective way of increasing the number of patients cured of malaria through higher ACT adherence rates.

This study also presents some evidence on the reasons malaria patients do not complete their medications. We find that patients who report earlier symptom resolution and believe they are cured earlier are substantially (15–50 percentage points) less likely to finish the medication. Moreover, the sticker interventions increased adherence primarily among this group of patients, which suggests that the short messages may have convinced patients not to rely entirely on their own symptoms and beliefs about cure in determining whether to finish their medication. We also show that patients who were unfamiliar with ACTs prior to the study were less likely to complete the medication. This suggests that perceptions of drug effectiveness also influence ACT adherence rates in this context.

Our results suggest that interventions successful in increasing adherence rates will need to convince patients to continue taking the medication even once symptoms have resolved, while also increasing patients' confidence in the effectiveness of the drugs. It is possible that the scale-up of malaria diagnostic testing could highlight for patients the imperfect connection between symptoms and malaria positivity. Higher rates of diagnostic testing could also enable patients to learn about the effectiveness of ACTs in treating the disease (Adhvaryu, 2014), though this will depend on the extent to which health workers comply with the results of the malaria diagnostic test in prescribing ACTs, which has varied considerably across different contexts (Odaga et al.).

Since 60–70% of patients are cured with only four doses of the drug, encouraging patients who are cured after four doses of ACTs to complete the six-dose treatment may result in “over-adherence,” where some people continue taking a drug that does not benefit them. Egan and Philipson (2014) suggest that many interventions targeting non-adherence may be inefficient if they also increase over-adherence. With malaria, however, the costs of adherence are very low (the drugs are relatively inexpensive, side effects are minimal, and the treatment is short), and the benefits are very high (under-treated malaria can be deadly and can have large externalities), so it is likely to still be socially beneficial to encourage full treatment compliance despite some over-adherence.

There are several limitations of this study. We cannot say precisely why the short messages were more effective in increasing adherence compared to the more detailed CAPSS/CAPSS-Information packages which contained much of the same information. The stickers may simply have been more visible or, because they only consisted of a single message, they may have highlighted for patients the importance of adherence. We also do not have sufficient data to determine how patients responded to different parts of the messages on the stickers: whether they were primarily influenced by the injunction to finish the medication or whether the reasons for finishing the medication were also important, though our evidence suggests that the salience of the adherence message may have been more relevant in this context. More research is needed to understand how the content and design of messages affects patients' beliefs and behaviors. Finally, our study was not powered to determine the impact of the interventions on malaria transmission in this context.

While our interventions had moderate impacts on adherence, they do help shed light on why people may be stopping their medication and what types of interventions might be successful in increasing adherence rates. Further research to better understand how people's beliefs about malaria illness and treatment are formed may enhance our understanding of why they are so difficult to change.

## Funding sources

This work was supported by the Clinton Health Access Initiative, the Bill and Melinda Gates Foundation and the Department for International Development.

## Figures and Tables

**Table A1:** Dosing regimen and prices of ACTs.

Dosage Groups	Number of Pills Per Dose	Dosing Schedule	Number of Treatment Days	Subsidized Price USH (Ugandan Shillings)	Subsidized Price (USD)
4 months-<3 years	1	2 X Day (Morning and Evening)	3 Days	200	0.09
3 years-<7 years	2			400	0.17
7 years-<12 years	3			600	0.26
12 years and above	4			800	0.35

Dosing regimen and prices of ACTs. The exchange rate in December 2010 was approximately 2250 USH to $1 USD.

**Table A2:** Loss to follow-up across treatment groups.

	Mean in Control Group	CAPSS	CAPSS-Info Only	“Malaria is NOT gone until…” message	“Don't Save Pills…” Message	Obs
	(1)	(2)	(3)	(4)	(5)	(6)
Follow Up Completed	0.963 (0.189)	−0.008 [0.019]	0.014 [0.020]	0.004 [0.021]	−0.044∗ [0.020]	1850
Follow Up Completed in 96 hours or less	0.928 (0.259)	−0.013 [0.039]	0.000 [0.026]	−0.019 [0.030]	−0.039 [0.022]	1850
Blisterpack Available at Follow-Up Survey	0.864 (0.343)	−0.069∗∗ [0.026]	0.052∗ [0.022]	0.006 [0.031]	0.03 [0.036]	1775

Column 1 shows the mean and standard deviation (in parentheses) in the control group for patients who started taking their medication. Columns 2–5 show the coefficients on dummies for each of the treatment groups with standard errors (clustered by shop) in square brackets. The regression controls for shop, day, ACT purchase number, and previous pack type fixed effects. Regressions also control for whether a free rapid diagnostic test (RDT) was offered, and an interaction of each pack type with a dummy for whether an RDT was offered. ^∗^p < 0.10, ^∗∗^p < 0.05, ^∗∗∗^p < 0.01.

**Table A3:** Robustness checks on the impact of packaging and messaging on adherence.

	Dependent Variable is Adhered (Completed All Medication)					
	(1)	(2)	(3)	(4)	(5)	(6)
A. CAPSS	−0.027 (0.040) [0.520] {0.570}	0.021 (0.073) [0.782] {0.878}	−0.028 (0.035) [0.453] {0.464}	−0.033 (0.039) [0.419] {0.428}	−0.071∗ (0.032) [0.061] {0.036}	−0.019 (0.032) [0.571] {0.538}
B. CAPSS INFO ONLY	−0.052 (0.057) [0.393] {0.486}	−0.027 (0.077) [0.737] {0.740}	−0.030 (0.032) [0.369] {0.350}	−0.044 (0.037) [0.277] {0.242}	−0.041 (0.032) [0.231] {0.262}	−0.021 (0.030) [0.508] {0.452}
C. “MALARIA IS NOT GONE UNTIL…” MESSAGE	0.056∗ (0.025) [0.058] {0.004}	0.078 (0.078) [0.351] {0.390}	0.041∗ (0.019) [0.062] {0.000}	0.058∗∗∗ (0.014) [0.003] {0.000}	0.078∗∗∗ (0.018) [0.003] {0.000}	0.075∗∗∗ (0.011) [0.000] {0.000}
D. “DON'T SAVE PILLS…” MESSAGE	0.046 (0.057) [0.447] {0.532}	0.072 (0.089) [0.438] {0.436}	0.048 (0.046) [0.327] {0.344}	0.052 (0.049) [0.321] {0.372}	0.080 (0.046) [0.118] {0.134}	0.067 (0.043) [0.162] {0.164}
Mean of Outcome in Control Group	0.623	0.584	0.648	0.631	0.599	0.638
P-value: C = D	0.786	0.942	0.889	0.898	0.975	0.839
R-squared	0.157	0.221	0.133	0.147	0.176	0.148
Number of Observations	1356	718	1755	1732	1480	1682

Only First Individual ACT Purchases	Yes	No	No	No	No	No
Only First Household ACT Purchases	No	Yes	No	No	No	No
Follow-Up Window	<=96 h	<=96 h	All	<=96 h	<=96 h	<=96 h
Includes Patients Who Didn't Start Medication	No	No	No	Yes	No	No
Only Those who Showed Blisterpack	No	No	No	No	Yes	No
Includes Additional Control Variables	No	No	No	No	No	Yes

Regression specifications are based on Equation (1). Columns 1 and 2 exclude ACT purchase number and previous pack type fixed effects. The additional control variables in Column 6 are age of patient, probability of a malaria episode in the household in the month prior to the baseline survey and whether the female household head had heard of ACTs prior to the study. Except where noted, sample is limited to those who started taking the medication and were visited for a follow-up survey within 96 h of ACT purchase. Standard errors are in parentheses and clustered at the shop level, p-values in square brackets. P-values using wild bootstrap clustered standard errors are in curly braces. ^∗^p < 0.10, ^∗∗^p < 0.05, ^∗∗∗^p < 0.01.

**Table A4:** Robustness of main results to limiting sample to patients not offered free RDT at drug shop.

Coefficient on:	By pack type			Pooled		
	Adhere	Doses	Tablets	Adhere	Doses	Tablets
	(1)	(2)	(3)	(4)	(5)	(6)
A. CAPSS	−0.037 (0.041) [0.396] {0.380}	0.099 (0.164) [0.560] {0.586}	0.395 (0.492) [0.445] {0.452}			
B. CAPSS Info Only	−0.052 (0.038) [0.213] {0.218}	0.260 (0.208) [0.247] {0.294}	0.709 (0.721) [0.354] {0.382}			
C. “Malaria is not gone until…” Message	0.052∗∗ (0.018) [0.023] {0.000}	−0.179 (0.156) [0.286] {0.298}	−0.635 (0.454) [0.200] {0.284}			
D. “Don't save pills…” Message	0.027 (0.040) [0.515] {0.506}	−0.075 (0.115) [0.532] {0.488}	−0.474 (0.393) [0.262] {0.248}			
E. CAPSS/CAPSS Info Only (A and B combined)				−0.042 (0.032) [0.226] {0.208}	0.181 (0.149) [0.260] {0.308}	0.587 (0.490) [0.266] {0.292}
F. Sticker Messages (C and D combined)				0.043∗ (0.023) [0.095] {0.068}	−0.128 (0.094) [0.213] {0.204}	−0.527 (0.329) [0.147] {0.140}

Mean of Dependent Variable	0.631	0.895	2.445	0.631	0.895	2.445
Test (C = D)	0.553	0.553	0.758			
Test (E = F)				0.002	0.040	0.040
R squared	0.183	0.214	0.256	0.178	0.209	0.253
Number of Obs	1321	1318	1318	1321	1318	1318

Regression specifications are based on Equation (1). Regressions with tablets as an outcome also include dosage group fixed effects. Sample is limited to those who were not offered an RDT, who started taking the medication and were visited for a follow-up survey within 96 h of ACT purchase. Standard errors are in parentheses and clustered at the shop level, p-values in square brackets. P-values using wild bootstrap clustered standard errors are in curly braces. ^∗^p < 0.10, ^∗∗^p < 0.05, ^∗∗∗^p < 0.01.

**Table A5:** Robustness of main results to assumptions about adherence rates among patients not showing medication blisterpack.

	*Dependent Variable: Adhered*		
	No Blisterpack =Adhered	No Blisterpack =Did Not Adhere	No Blisterpack =Blisterpack Adherence Rate
	(1)	(2)	(3)
A. CAPSS	−0.030 (0.037) [0.439] {0.456}	−0.109∗∗∗ (0.032) [0.009] {0.012}	−0.071 (0.040) [0.112] {0.092}
B. CAPSS INFO ONLY	−0.058 (0.034) [0.128] {0.168}	−0.012 (0.035) [0.727] {0.680}	−0.049 (0.035) [0.204] {0.216}
C. “MALARIA IS NOT GONE UNTIL…” MESSAGE	0.053∗∗ (0.021) [0.035] {0.036}	0.057∗ (0.026) [0.064] {0.002}	0.058∗∗ (0.025) [0.049] {0.000}
D. “DON'T SAVE PILLS…” MESSAGE	0.045 (0.038) [0.272] {0.266}	0.058 (0.037) [0.153] {0.170}	0.052 (0.037) [0.195] {0.186}
Mean of Dependent Variable	0.654	0.516	0.600
P-value: C = D	0.849	0.970	0.893
R-squared	0.172	0.221	0.176
Number of Observations	1298	1298	1299

We assume in Column 1 that everyone who did not show their blisterpack adhered, in Column 2 that people who did not show their blisterpack did not adhere, and in Column 3 that the adherence rate among those who did not show their blisterpack is the same as the adherence rate among those who did show their blisterpack (separately by each pack type). Regressions include shop, day, ACT purchase number, and previous pack type fixed effects. Sample is limited to those who were not offered a free rapid diagnostic test for malaria, who started taking their medication and who were visited for a follow-up survey within 96 h of ACT purchase. Standard errors are in parentheses and clustered at the shop level, p-values in square brackets. P-values using wild bootstrap clustered standard errors are in curly braces. ^∗^p < 0.10, ^∗∗^p < 0.05, ^∗∗∗^p < 0.01.

**Table A6:** Beliefs about adherence at endline survey by sticker message received.

	Respondent believed that non-adherence could lead to patient not getting better, feeling sicker, or death	Respondent believed that non-adherence could be harmful for household or community (resistance/transmission)	Respondent “agreed” or “strongly agreed” that the number of malaria pills depends on how sick the patient feels	Respondent “agreed” or “strongly agreed” that it was important to take the treatment for 3 days
	(1)	(2)	(3)	(4)
A. Ever Received CAPSS	−0.012	0.032	−0.005	0.066∗∗∗
	(0.021)	(0.032)	(0.036)	(0.018)
	[0.579]	[0.341]	[0.894]	[0.006]
	{0.634}	{0.390}	{0.926}	{0.006}
B. Ever Received CAPSS INFO ONLY	0.018	0.063	−0.069	0.019
	(0.018)	(0.047)	(0.037)	(0.024)
	[0.348]	[0.222]	[0.101]	[0.446]
	{0.382}	{0.236}	{0.110}	{0.480}
C. Ever Received “MALARIA IS NOT GONE UNTIL…” MESSAGE	0.018	0.037	−0.031	0.038
	(0.025)	(0.038)	(0.035)	(0.027)
	[0.499]	[0.351]	[0.405]	[0.193]
	{0.462}	{0.324}	{0.390}	{0.182}
D. Ever Received “DON'T SAVE PILLS…” MESSAGE	0.002	0.047	−0.006	0.035
	(0.025)	(0.037)	(0.026)	(0.032)
	[0.938]	[0.231]	[0.833]	[0.302]
	{0.056}	{0.332}	{0.232}	{0.952}
Mean of Outcome in Control Group	0.921	0.320	0.312	0.837
P-value: C = D	0.553	0.832	0.557	0.949
R-squared	0.019	0.018	0.025	0.019
Number of Observations	1091	1091	1034	1036

Notes: Data is from endline survey where only one respondent was interviewed per household. The outcomes in Columns 1 and 2 are binary variables that indicate whether the respondent gave any of those responses to an open-ended question about what can happen if a person does not finish their malaria medication and saves the pills for later. The outcomes in Columns 3 and 4 are binary variables indicating whether the respondent said “Strongly Agree” or “Agree” on a 5-point Likert scale to the statements described. Regressions include shop fixed effects and control for whether anyone in the household was ever offered an RDT. Sample is limited to households who ever purchased an ACT through the study. Standard errors are in parentheses and clustered by shop, p-values are in square brackets. P-values using wild bootstrap clustered standard errors are in curly braces. ^∗^p < 0.10, ^∗∗^p < 0.05, ^∗∗∗^p < 0.01.

**Table A7:** Impact of packaging and messaging on adherence and medication-taking by patient/caregiver English literacy.

Coefficient on:	Adhere	Doses	Tablets
	(1)	(2)	(3)
A. CAPSS	0.008 (0.067) [0.905] {0.894}	0.049 (0.226) [0.833] {0.848}	0.353 (0.688) [0.622] {0.628}
B. CAPSS Info Only	−0.008 (0.053) [0.888] {0.890}	0.128 (0.204) [0.547] {0.524}	0.423 (0.671) [0.546] {0.576}
C. “Malaria is not gone until…” Message	0.021 (0.045) [0.657] {0.624}	−0.039 (0.151) [0.804] {0.818}	−0.205 (0.493) [0.688] {0.718}
D. “Don't save pills…” Message	0.014 (0.047) [0.769] {0.724}	−0.022 (0.155) [0.889] {0.866}	−0.241 (0.490) [0.636] {0.654}
E. Patient/Caregiver Can Read English	0.109∗∗ (0.039) [0.024] {0.040}	−0.205 (0.117) [0.117] {0.140}	−0.466 (0.378) [0.253] {0.228}
F. CAPSS X Patient/Caregiver Can Read English	−0.093 (0.081) [0.283] {0.222}	0.104 (0.195) [0.609] {0.648}	0.086 (0.601) [0.889] {0.862}
G. CAPSS Info Only X Patient/Caregiver Can Read English	−0.107 (0.097) [0.299] {0.312}	0.325 (0.247) [0.225] {0.208}	0.714 (0.861) [0.431] {0.460}
H. “Malaria is not gone until…” Message X Patient/Caregiver Can Read English	0.072 (0.099) [0.484] {0.522}	−0.316 (0.236) [0.218] {0.246}	−0.965 (0.691) [0.200] {0.216}
I. “Don't save pills…” Message X Patient/Caregiver Can Read English	0.030 (0.063) [0.651] {0.656}	−0.119 (0.202) [0.571] {0.616}	−0.528 (0.754) [0.503] {0.562}
Mean of Outcome in Reference Group (Control, No English)	0.601	0.936	2.411
R squared	0.195	0.224	0.263
Number of Observations	1319	1316	1316

The ability to read English is defined at the patient level for those aged 12 and above. For patients below the age of 12, they are defined as being able to read English if either the patient or the caregiver can read English. Regression specifications were based on Equation (1). Sample is limited to those who were not offered an RDT, who started taking the medication and were visited for a follow-up survey within 96 h of ACT purchase. Standard errors are in parentheses and clustered at the shop level, p-values in square brackets. P-values using wild bootstrap clustered standard errors are in curly braces. ^∗^p < 0.10, ^∗∗^p < 0.05, ^∗∗∗^p < 0.01.

**Table A8:** Understanding of dosing instructions on control and CAPSS packs.

	Number of Days	Number of Pills Per Dose	Time of Day
	A. Control Pack (Adult Dose)		
Correct	33.7%	61.1%	46.2%
Wrong	19.1%	35.1%	35.3%
Didn't Mention It	44.1%	0.84%	15.6%
Don't Know	3.00%	3.00%	2.96%
	B. CAPSS Pack (Adult Dose)		
Correct	33.4%	60.0%	46.4%
Wrong	18.1%	36.7%	35.7%
Didn't Mention It	46.0%	0.84%	15.4%
Don't Know	2.51%	2.56%	2.52%

Respondents were the female head of household, if available. At the endline survey, the surveyor showed the respondent each adult dose package (Control and CAPSS) separately and asked her to say how she would take the medication. Responses were not prompted. ‘Didn't Mention It’ is for people who didn't mention that particular aspect of the dosing regimen.

**Table A9:** Cost of averted infection using sticker messages.

	Proportion of Patients Testing Malaria Positive		
	100%	74%	67%
4-Dose 28-day Cure Rate: 71%^a^	$2.63	$3.56	$3.93
6-Dose 28-Day Cure Rate: 81%			
4-Dose 28-day Cure Rate: 61%^b^	$0.82	$1.11	$1.23
6-Dose 28-Day Cure Rate: 93%			
4-Dose 28-day Cure Rate: 76%^c^	$1.32	$1.78	$1.96
6-Dose 28-Day Cure Rate: 96%			

We assume that patients who receive the targeted sticker messages have an adherence rate of 69.5% while patients who receive the standard ACT package have an adherence rate of 63.8% (see Table 2, Column 4). Patients who do not adhere are assumed to take 4 doses of the medication instead of the recommended 6 doses. The cost of adding a sticker to the package is $0.015. Positivity rates are based on our measures of the sub-sample who were randomly tested (67%) and among the sub-sample under age five who were randomly tested (74%).

a Cure rates are from Vugt et al. (1999) and are not PCR-corrected to distinguish new from recurrent infections.

b PCR-corrected cure rates are from Makanga et al. (2006) and based on Intention-to-treat (all patients not available at 28-days were assumed as treatment failures).

c PCR-corrected cure rates are from Makanga et al. (2006) and based on evaluable population (patients available at 28-day follow-up).

## Appendix A Theoretical Model

In this section we present a simple two period model of the adherence decision. In period one a patient is hit with an illness shock that he believes is malaria with some probability *p*_1_, and begins taking malaria medication. In period two the patient decides whether to finish taking the pills or to stop treatment. The patient faces a tradeoff between the benefits of being cured of the disease and the costs of adhering to the medication. This tradeoff is mediated by the patient's perceived likelihood that he continues to suffer from the illness in the second period, the probability that the illness is actually malaria, and his belief in the effectiveness of the medication in treating malaria. We consider the predictions of the model under several simplified cases in order to provide intuition of how these different factors influence adherence in this framework.

### Appendix A.1 Definitions

We begin by defining the following terms:

• *s*_2_ denotes the severity of illness in period two after the patient has taken a few doses of the medication. We assume that illness severity in period two is independent of adherence behavior as 90% of patients in our sample took the first two doses of the medication on time.
• *π*_2_ is the probability the patient assigns to continuing to have the illness (either malaria, or some other disease) in period two. We assume that beliefs about the probability of still having the illness are a function of illness severity in period two (*π*_2_ = *f*(*s*_2_)) and that the perceived probability increases with symptom severity in period two (i.e. *π*^′^_2_(*s*_2_) > 0).
• *λ*_1_ is the probability the patient assigns in period one to the effectiveness of the drug in treating malaria and *λ*_2_ is the patient's updated probability in period two that the drug is effective in treating malaria.
• *p*_1_ is the patient's perceived probability in period one (prior to beginning treatment) that the illness he is suffering from is malaria and *p*_2_ is the patient's updated probability in period two that the illness he was suffering from (and potentially continuing to suffer from) is malaria.
• *c* is the cost of continuing to take the treatment in period two. The costs include possible side effects of the drugs, the effort to remember to take the drugs, and the opportunity cost of consuming pills that could otherwise be used to treat future malaria episodes. We assume that the cost of adhering to the treatment is the same regardless of whether the person is, in fact, still sick with the illness.
• *U*^*H*^ is the utility that a person gets from being healthy, while *U*^*S*^ is the utility that a person gets from being sick (which we normalize so that *U*^*S*^ = 0). The utility of being healthy includes factors such as increased productivity and wages as well as the intrinsic value of being healthy. We also include within *U*^*H*^ the patient's perceptions of the benefits to society of his being cured of malaria (less likelihood of malaria transmission, lower probability that the parasite will develop resistance to the drug). For simplicity, we assume the utility of being sick *U*^*S*^ is the same regardless of whether the patient is suffering from malaria or some other disease.

### Appendix A.2 Patient Decision-Making

We first consider the case in which the patient has no uncertainty that the illness is malaria (i.e. *p*_1_ = *p*_2_ = 1) and believes that the drugs are fully effective in treating the disease (i.e. *λ*_1_ = *λ*_2_ = 1). In period one, the patient is hit with an illness shock he believes is malaria and begins taking ACTs. After having taken the first two or three doses of the medication in period one the patient has two possible actions in period two, *a*ε*A*, *N*: (1) Continue to finish all the medications (i.e. adhere to treatment guidelines) *a* = *A* or (2) Stop taking the medication (i.e., not adhere to treatment guidelines) *a* = *N*. The action decision in period two is based partly on the perceived likelihood of still having malaria (*π*_2_), which is a function of the severity of the symptoms (*s*_2_) that the patient is experiencing in period two.

Then the expected utility of adhering *V*^*A*^(*π*) is as follows:

(A.1) $$V^{A}\left(\pi_{2}\right)=\pi_{2}\;*\;\left[U^{H}-c\right]+\left[1-\pi_{2}\right]\;*\;\left[U^{H}-c\right]$$

(A.2) $$V^{A}\left(\pi_{2}\right)=U^{H}-c$$

The expected utility of not adhering is:

(A.3) $$V^{N}\left(\pi_{2}\right)=\left[1-\pi_{2}\right]\;*\;U^{H}$$

By finishing the medication the patient pays the cost *c* in order to ensure that he will be healthy, regardless of whether or not he is still suffering from malaria in period two. If the patient chooses not to finish the medication he avoids the cost *c*, but assumes some risk that he is not fully cured of the disease and may continue to suffer from malaria (either from the current infection or a future recrudescence of the infection). The patient will adhere to the treatment if the expected value of adhering to the treatment exceeds the expected value of not adhering to the treatment:

(A.4) $$V^{A}\left(\pi_{2}\right)-V^{N}\left(\pi_{2}\right)>0$$

(A.5) $$U^{H}-c-\left[1-\pi_{2}\right]\;*\;U^{H}>0$$

(A.6) $$\pi_{2}\left(s_{2}\right)>\frac{c}{U^{H}}$$

This implies that a patient will adhere if the belief that he continues to suffer from malaria in period two exceeds some threshold value of *c*/*U*^*H*^. Patients are thus more likely to adhere when symptom severity is relatively high in period two, when the costs of adhering are low (few side effects, low value of saved pills, etc.) and when the benefit to being healthy is high (see Fig. 1A).

### Appendix A.3 Uncertainty about Drug Effectiveness

We now turn to the case in which the patient still believes the illness is malaria (*p*_1_ = *p*_2_ = 1) but is unsure whether the drugs he is taking are effective in treating malaria. The patient therefore updates the probability that the drugs are effective in period two based on his priors in period one that the drugs were effective and the severity of his symptoms in period two *λ*_2_ = *f*(*λ*_1_, *s*_2_). Since the patient is certain that the illness is malaria, holding constant his priors about the drug's effectiveness, the greater the severity of the illness in period two, the more likely he is to believe that the drug is not effective in treating malaria. Thus, we assume that *λ*^′^_2_(*s*_2_) < 0. The expected utility of adhering *V*^*A*^(*π*_2_, *λ*_2_) is as follows:

(A.7) $$V^{A}\left(\pi_{2,}\lambda_{2}\right)=\pi_{2}\;*\left[\lambda_{2}U^{H}+\left[1-\lambda_{2}\right]*U^{S}-c\right]+\left[1-\pi_{2}\right]\;*\left[U^{H}-c\right]$$

(A.8) $$V^{A}\left(\pi_{2},\lambda_{2}\right)=\pi_{2}\lambda_{2}U^{H}+\left[1-\pi_{2}\right]\;*\;U^{H}-c$$

The expected utility of not adhering is the same as in the previous section:

(A.9) $$V^{N}\left(\pi_{2}\right)=\left[1-\pi_{2}\right]\;*\;U^{H}$$

Once again, the patient will adhere to the treatment if the expected value of adhering to the treatment exceeds the expected value of not adhering to the treatment:

(A.10) $$V^{A}\left(\pi_{2},\lambda_{2}\right)-V^{N}\left(\pi_{2}\right)>0$$

(A.11) $$\pi_{2}\lambda_{2}U^{H}+\left[1-\pi_{2}\right]*U^{H}-c-\left[1-\pi_{2}\right]\;*\;U^{H}>0$$

(A.12) $$\pi_{2}\lambda_{2}U^{H}-c>0$$

(A.13) $$\pi_{2}\left(s_{2}\right)\lambda_{2}\left(\lambda_{1},s_{2}\right)>\frac{c}{U^{H}}$$

As before, the likelihood of adhering increases with the utility of being healthy and decreases with the cost of adhering. However, since *π*^′^_2_(*s*_2_) > 0 and *λ*^′^_2_(*s*_2_) < 0, there is a non-linear relationship between the probability of adhering and the severity of the disease in period two (see Fig. 1B). At low symptom severities in period two, patients perceive the drug to be very effective in treating malaria, but are less likely to believe that they are still suffering from malaria and so the expected value of finishing the medication is low. At high symptom severities, patients are more likely to believe that they still have malaria, but the perceived probability that the drug is effective is low, so the expected utility of adhering is again low. The expected utility of adhering is therefore maximized at intermediate levels of illness severity in the second period.

### Appendix A.4 Uncertainty About Malaria Diagnosis

Finally, we consider the case where the patient is uncertain about whether the illness he is suffering from is malaria in period one, but believes that the drugs are effective in treating malaria (*λ*_1_ = *λ*_2_ = 1). In this case, he updates his beliefs about the likelihood of the illness being malaria in period two (*p*_2_) based on his beliefs that the illness was malaria in period one and the severity of his symptoms in period two (*p*_2_ = *f*(*p*_1,_*s*_2_)). Since the patient is confident that the drug treats malaria, holding constant his priors about the likelihood that the illness was malaria, the greater the severity of the symptoms in period two, the more likely he is to believe that his illness was not malaria to begin with. Thus we assume that *p*^′^_2_(*s*_2_) < 0. The patient will continue taking the medication in period two if the utility of adhering exceeds the expected utility of not adhering to the treatment:

(A.14) $$V^{A}\left(\pi_{2},p_{2}\right)-V^{N}\left(\pi_{2}\right)>0$$

(A.15) $$\pi_{2}p_{2}U^{H}+\left[1-\pi_{2}\right]*U^{H}-c-\left[1-\pi_{2}\right]\;*\;U^{H}>0$$

(A.16) $$\pi_{2}p_{2}U^{H}-c>0$$

(A.17) $$\pi_{2}\left(s_{2}\right)p_{2}\left(p_{1},s_{2}\right)>\frac{c}{U^{H}}$$

Thus the likelihood of adhering increases with the patient's perceived probability that he is still suffering from the illness (*π*_2_) and his perceived probability in period two that the illness is actually malaria (*p*_2_). Since *π*^′^_2_(*s*_2_) > 0 and *p*^′^_2_(*s*_2_) < 0, as in Section A3, there is a non-linear relationship between the probability of adhering and the severity of the disease in period two. At low symptom severities, patients are more likely to believe that the illness is malaria (since the drug was effective in treating the disease), but are also more likely to believe that they are cured, so the expected value of finishing the medication is low. At very high symptom severities, while patients are more likely to believe that they still have the illness, the perceived probability that the illness is actually malaria is low, so the expected utility of adhering is again low. As in Section A3, the expected utility of adhering is therefore maximized at intermediate levels of illness severity in the second period.

### Appendix A.5 Model Implications

The model assumes that adherence to ACTs is a function of patients' beliefs about the likelihood of the illness being malaria, their beliefs about the effectiveness of the drug in treating malaria, and also their beliefs about whether they are cured of the illness mid-way through the treatment course. In period one, patients have some prior beliefs about the likelihood of the illness being malaria and the likelihood that the drug is effective in treating malaria. In period two, the patient updates these beliefs based on the severity of his symptoms and decides whether to continue taking the drug.

If the patient feels relatively healthy in period two, he is likely to conclude that he is cured, regardless of whether he believes the illness was malaria or some other disease. In this case uncertainty about the diagnosis and about the effectiveness of the drug have less impact on adherence rates. If the patient feels very ill in period two, he is more likely to believe that he is not cured of the illness but is also likely to conclude that either the illness was not malaria to begin with (if in period one he believed that the drugs were quite effective in treating malaria), or that the drugs are not effective in treating malaria (if instead he was confident, in period one, that the illness was malaria). Thus, uncertainty about the diagnosis and about the effectiveness of the drugs in treating malaria both result in lower adherence at high symptom severities.

## Footnotes

2 A sub-therapeutic dose of ACTs can kill all sensitive parasites while allowing the more resistant parasites to survive (thus “selecting” for resistant parasites) (White et al., 2009).

3 The “28-day cure rate” is defined as the clearance of asexual parasites within 7 days of beginning treatment without recrudescence (reappearance) in 28 days. In highly endemic areas, it is possible for a person to get re-infected with malaria within this time frame. Thus, in some cases, genetic analysis is used to distinguish between a recurrence of the same infection and a new infection so that the latter are not counted as treatment failures.

4 For example, according to the World Health Organization (WHO), “in endemic regions, some semi-immune malaria patients could be cured using an incomplete dose or treatment regimens that would be unsatisfactory in patients with no immunity. In the past, this had led to different recommendations for patients considered as semi-immune and those considered as non-immune. This practice is no longer recommended. A full treatment course with a highly effective ACT is required whether or not the patient is considered to be semi-immune.” (WHO, 2010b).

5 We limit our empirical analyses to those who begin treatment.

6 On May 13, 2011 the Ministry of Health of Uganda confirmed a fatal case of Ebola Haemorrhagic Fever in Luwero District. As a result, the study was halted for approximately a month between May 18, 2011 and June 16, 2011. While surveying and project research stopped during this time, we ensured a steady supply of ACTs at shops during this period. We find no evidence that this break in study implementation affected adherence rates.

7 Drug shops were selected from a list of licensed shops provided by the Luwero District Area Drug Inspector. Shops were selected based on shop owner qualifications, length of time the shop had been in business, daily customer traffic and operating days/hours. We selected shops that were well qualified and established and that had sufficient customer traffic to reach the desired sample size in a five month period but were not so large that the traffic would be unmanageable for our survey team.

8 No restrictions were placed on the number of times the card could be used in order to avoid intra-household rationing. However, the project had a limited budget and could not accomodate excessive purchases caused, for example, by hoarding. Hoarding did not turn out to be a serious problem and our approach to this was informal. In the limited cases in which a household seemed to be purchasing an excessive number of ACT doses (34 households, or 3 percent of households that purchased an ACT, bought more than 6 doses of ACTs over the course of the study), we would have a surveyor visit the household and inquire about the health of the members, reminding the household head that the cards were only to be used for patients in the household who were currently sick. This approach worked very well throughout the study.

9 This is the same number of households that were found in the census activity. No households declined to participate in the study.

10 We refer to “shop owners” throughout the paper loosely to refer to either the shop owner him/herself or to the shop attendants (who man the shop but might not be owners). All shop personnel were trained on ACT dosing and prescribing.

11 In early July 2010, the probability of follow up was increased to 85 percent because we found that we had the survey team capacity to do additional follow up surveys. As this was the last month of the study, it did not increase the overall probability of follow-up among our sample of ACT-purchasing households by much. Overall, of the 2516 patients purchasing ACTs over the course of the study, 76.8 percent of them were members of households that were assigned to receive a follow-up visit.

12 This was a 95% subsidy on the retail price, and was chosen because it was the expected price of the drugs under the Affordable Medicines Facility malaria (AMFm), a global pilot ACT subsidy program (Gelband et al., 2004; Laxminarayan and Gelband, 2009).

13 The first two doses of ACT are to be taken 8 h apart and the remaining doses should be taken every 12 h, generally in the morning and evening so that the entire course should take 56 h from initial dose to completion.

14 The CAPSS study took place between August 2007 and May 2010 in five districts. CAPSS was completed six months before this study took place and was not in (or near) Luwero district.

15 Although this message may not be true for some patients, as noted above, the WHO recommendation is for all patients to finish medication in order for them to ensure parasite clearance and there is a general consensus that people are more likely to respond to simple messages rather than more complex, nuanced messaging (Cutler; Randolph and Viswanath, 2004).

16 An additional treatment arm was also included in the study with a sticker that provided the actual (non-subsidized) price of the medicines. However, due to budgetary reasons, this treatment had to be phased out early in the study before we obtained a reasonable sample size. We control for this treatment arm in all analyses but do not present the results.

17 In practice, 80% (1632/2040) of individuals who purchased an ACT did so only once and 43% (480/1107) of households who bought an ACT did so only once.

18 According to the survey team, the main reason that the CAPSS blister pack was slightly less likely to be available for inspection was because some households were using the CAPSS package insert to help start fires. We explore the robustness of our results to various assumptions about adherence among those who did not show their blister packs in Appendix Table A5.

19 We also checked for balance across characteristics of ACT purchasers who were assigned to a follow-up visit and those who were not. Only two variables were significantly different (patients and household heads of those assigned to receive a follow-up visit were likely to be younger than those not assigned to receive a follow-up visit) but the magnitude of the differences were on the order of 1–2 years (Results not shown).

20 We see a similar pattern if we look, instead, at patients who at baseline said that, if money were no object, they would prefer to take ACTs (compared to those who didn't), or at patients who said that they believed ACTs were the most effective drug for treating malaria in adults (compared to those who mentioned other drugs). While the latter is perhaps the most direct measure of beliefs about effectiveness, we don't have this variable for the entire sample–only for the sample who mentioned ACTs as a drug they have heard of. All of these measures yield similar results.

21 To our knowledge, there are no studies that provide evidence on concurrent symptoms and lingering parasite load for ACT-takers, but it is reasonable to assume that the resolution of symptom severity is not a good indicator of parasite clearance, since ACTs are known to begin relieving symptoms quickly (White et al., 1999; Makanga et al., 2011).

22 While the instructions given at the shop may have been insufficient, it is likely that they were the best instructions patients would get in this context. This is because our shop attendants were provided with special training in ACT administration, were among the largest and most professional shops in these areas, and were working side by side with our study team throughout the project.

23 It is important to note, however, that social marketing campaigns also have the objective of increasing uptake of products which we do not examine in this study.
